# Dynamics of mRNA fate during light stress and recovery: from transcription to stability and translation

**DOI:** 10.1111/tpj.16531

**Published:** 2023-11-10

**Authors:** Aaron B. Smith, Diep R. Ganguly, Marten Moore, Andrew F. Bowerman, Yoshika Janapala, Nikolay E. Shirokikh, Barry J. Pogson, Peter A. Crisp

**Affiliations:** ^1^ Research School of Biology The Australian National University Canberra Australian Capital Territory 2601 Australia; ^2^ CSIRO Synthetic Biology Future Science Platform Canberra Australian Capital Territory 2601 Australia; ^3^ Department of Biology University of Pennsylvania Philadelphia Pennsylvania 19104 USA; ^4^ Department of Biochemistry and Molecular Biology, Monash Biomedicine Discovery Institute Monash University Clayton Victoria 3800 Australia; ^5^ The John Curtin School of Medical Research, The Shine‐Dalgarno Centre for RNA Innovation The Australian National University Canberra Australian Capital Territory 2601 Australia; ^6^ School of Agriculture and Food Sciences The University of Queensland Brisbane Queensland 4072 Australia

**Keywords:** mRNA stability, translation, light stress, recovery, *Arabidopsis thaliana*

## Abstract

Transcript stability is an important determinant of its abundance and, consequently, translational output. Transcript destabilisation can be rapid and is well suited for modulating the cellular response. However, it is unclear the extent to which RNA stability is altered under changing environmental conditions in plants. We previously hypothesised that recovery‐induced transcript destabilisation facilitated a phenomenon of rapid recovery gene downregulation (RRGD) in *Arabidopsis thaliana* (Arabidopsis) following light stress, based on mathematical calculations to account for ongoing transcription. Here, we test this hypothesis and investigate processes regulating transcript abundance and fate by quantifying changes in transcription, stability and translation before, during and after light stress. We adapt syringe infiltration to apply a transcriptional inhibitor to soil‐grown plants in combination with stress treatments. Compared with measurements in juvenile plants and cell culture, we find reduced stability across a range of transcripts encoding proteins involved in RNA binding and processing. We also observe light‐induced destabilisation of transcripts, followed by their stabilisation during recovery. We propose that this destabilisation facilitates RRGD, possibly in combination with transcriptional shut‐off that was confirmed for *HSP101*, *ROF1* and *GOLS1*. We also show that translation remains highly dynamic over the course of light stress and recovery, with a bias towards transcript‐specific increases in ribosome association, independent of changes in total transcript abundance, after 30 min of light stress. Taken together, we provide evidence for the combinatorial regulation of transcription and stability that occurs to coordinate translation during light stress and recovery in Arabidopsis.

## Significance Statement

We provide a method for administering the transcriptional inhibitor, cordycepin, to mature Arabidopsis leaves. We study changes in mRNA stability and translation during light stress and recovery.

## INTRODUCTION

Responses to stress have been the subject of much research; however, the post‐stress recovery phase has been understudied and is likely to be equally complex and dynamic. Often, the molecular changes that occur at the onset of stress are protective in nature and divert resources away from growth and reproduction. Recovery, therefore, serves the purpose of resetting these protective processes in order to maximise growth potential. A balance is required between such resetting and maintaining the expression of acclimatory proteins (Bäurle & Trindade, 2020; Crisp et al., 2016). Indeed, it may be inefficient to maintain protective mechanisms indefinitely as demonstrated by the growth penalty incurred by some stress‐tolerant plant lines (Mickelbart et al., 2015). For instance, activity of the K^+^ efflux antiporter 3 (KEA3) was found to be important for relaxation of the energy‐dependent portion of non‐photochemical quenching (qE) following light stress (Armbruster et al., 2014). Increasing *KEA3* expression resulted in faster qE relaxation, in turn resulting in enhanced photosystem II efficiency under fluctuating light (Armbruster et al., 2016), which can influence CO_2_ fixation and biomass accumulation (Kromdijk et al., 2016). This example highlights the potential in optimising stress recovery in food crops.

Transcriptome resetting has been characterised during recovery from multiple stressors. For example, sulphur starvation in Arabidopsis led to the upregulation of many sulphur metabolism‐associated genes, which returned to pre‐stress levels within 3 h of resupply (Bielecka et al., 2014). Recovery of rice from phosphate starvation occurred within one day of resupply, although full recovery took 31 days in line with re‐establishing the original phosphate content (Secco et al., 2015). On the contrary, rice recovered from submergence within hours (Locke et al., 2018). We previously highlighted that the Arabidopsis transcriptome is rapidly reset following light stress, whereby stress‐induced mRNAs are downregulated to pre‐stress levels within 30 min; a phenomenon termed rapid recovery gene downregulation (RRGD) (Crisp et al., 2017). Of note, the observation that RRGD loci exhibited far shorter half‐lives during recovery compared with steady‐state measurements (Narsai et al., 2007), suggesting transcript‐specific destabilisation. The ability to rapidly alter the composition of the transcriptome gains particular importance during stress, when factors such as dehydration, heat and oxidative stress can cause extensive cellular damage. While transcription can be adjusted to produce stress‐associated transcripts, post‐transcriptional changes, including modulating mRNA stability, are likely to permit faster changes in abundance; a valuable feature when a rapid response is required (Pérez‐Ortín et al., 2007). Subtle shifts in stability can also greatly affect the ultimate number of mRNA molecules per cell over time (Ross, 1995). It is worth noting that recovery is more than just the resumption of pre‐stress gene regulation, as a diversity of recovery‐induced gene expression programmes is evident (Crisp et al., 2017; Yeung et al., 2018). For example, the downregulation of stress‐induced transcripts during recovery is accompanied by the upregulation of distinct genes, the products of which may play roles in recovery. This includes genes encoding RNA decay factors, such as the deadenylase‐encoding *CAF1* (Crisp et al., 2017). Whether these transcripts are translated proportional to their upregulation is unclear, but it may indicate a rapid means of re‐establishing pre‐stress cellular conditions.

There is increasing evidence that mRNA stability is altered during plant stress responses. Under high salinity, *N* ^6^‐methyladenosine promotes stabilisation of transcripts encoding salinity‐associated proteins (Kramer et al., 2020). During cold stress, coordinated changes in DNA transcription and mRNA stability culminated into faster changes in overall expression of cold‐responsive genes (Arae et al., 2017). Transcript degradation during stress appears to utilise the 5′ → 3′ RNA decay pathway through the activity of decapping proteins and their enhancers (DCP1, DPC2, DCP5 and VCS), and exoribonuclease (XRN) 4. For example, 5′ → 3′ RNA decay has been implicated in thermal acclimation (Merret et al., 2013; Nguyen et al., 2015), photomorphogenesis (Jang et al., 2019), pathogen resistance (Yu et al., 2019), ABA signalling (Wawer et al., 2018) and osmotic stress (Soma et al., 2017; Xu & Chua, 2012). Inhibition of the nuclear XRNs, resulting in RNA polymerase II read‐through into stress‐associated genes, has also been associated with drought tolerance (Crisp et al., 2018; Estavillo et al., 2011). These studies highlight the biological importance and potential specificity of the RNA decay machinery.

Translation is intimately tied with RNA decay in both cooperative and antagonistic ways (Roy & Jacobson, 2013). Competition for the 5′ cap can be a key determinant of mRNA stability, and high ribosome density and translation speed can commonly be an indicator of elevated stability (Chan et al., 2018; Hanson et al., 2018; Schwartz & Parker, 2000), although the inverse relationship has also been observed (Dave et al., 2023; Tuck et al., 2020). Conversely, co‐translational decay can degrade transcripts bound to ribosomes that are stalled or elongating at a slowed rate, thereby permitting ribosome recycling and ensuring the fidelity of produced peptides (Hou et al., 2016; Merret et al., 2015; Yu et al., 2016). We previously provided evidence for elevated co‐translational decay of light‐induced transcripts in Arabidopsis during recovery (Crisp et al., 2017). Notably, changes in co‐translational decay can also occur over plant development to influence translation in a transcript‐specific manner (Carpentier et al., 2020). Preferential translation may also permit a cell to make rapid changes in protein levels independently of transcription (Muñoz & Castellano, 2012). In order to prioritise resources during adverse conditions, translation is often globally repressed with the exception of a subset of transcripts required for stress responses (Chen et al., 2021; Juntawong et al., 2014). The repression of translation can involve degradation or transcriptional repression of genes encoding ribosomal proteins or translational factors (Munchel et al., 2011; Sheikh et al., 1999); however, a more rapid response is facilitated through dissociation of transcripts from initiation factors and ribosomes (Bresson et al., 2020; Liu & Qian, 2014). In many cases, the disassembly of polysomes is associated with the formation of cytoplasmic foci called stress granules, which store complexes of translationally inert mRNAs, translation initiation factors and other RNA‐binding proteins, thereby preventing their shuttling to, and degradation at, processing bodies (Chantarachot & Bailey‐Serres, 2018). Upon removal of the stress, transcriptional upregulation of the translation machinery occurs, likely to resume pre‐stress protein production (Yeung et al., 2018). This may be aided by the release of transcripts encoding ribosomal proteins from stress granules. For example, Arabidopsis heat shock proteins promote the disassembly of stress granules following heat stress, allowing stored mRNAs encoding initiation factor complexes to commence translation (Merret et al., 2017).

We hypothesise that mRNA stability and translation are combinatorially modulated during recovery from light stress, which contributes to the resetting of protective mechanisms by shaping the cellular RNA pool available for translation. Previously, we were not able to assess whether the stability of individual transcripts shifted between unstressed, stressed and recovery using conventional methods. An ongoing challenge with examining mRNA stability during stress is delineating changes in stability from changes in transcription. Accurate measurements of RNA decay require either cessation of transcription, through the use of transcriptional inhibitors, or *in vivo* labelling of mRNAs. The treatment of plants undergoing stress is limited by the nature and duration of the stress being examined. For example, delivery of RNA analogues via root feeding requires at least 1 h (Szabo et al., 2020), which is unsuitable for measurements with short‐term, transient stresses such as high light in which the transcriptome changes within minutes (Vogel et al., 2014). Previous attempts to model RNA half‐life in the absence of these treatments have had some success, but were heavily limited to upregulated transcripts that displayed stepwise reductions in abundance (Crisp et al., 2017).

To address the aforementioned shortcomings in prior reports, we established a method to perform transcriptional inhibition in leaves of mature soil‐grown plants using syringe infiltration of the transcriptional inhibitor, cordycepin. This enabled us to compare genome‐wide changes in mRNA stability between stress and recovery conditions *in situ*. We paired these with measurements of precursor mRNA (pre‐mRNA) levels, as a proxy for transcriptional changes, and with translation rates, by profiling ribosome‐associated mRNA during light stress and recovery. This combinatorial strategy revealed that RRGD was facilitated by an interplay between regulation of transcription and mRNA stability. While total mRNA abundance tracked with polysome‐associated RNA levels (i.e. translation), this relationship was uncoupled during late light stress and early recovery, suggesting the occurrence of translational re‐organisation.

## RESULTS

### Locus‐specific changes in transcription are evident during light stress and recovery

To determine whether transcriptional changes contribute to RRGD, we quantified changes in both pre‐mRNA and mRNA for three RRGD genes (Crisp et al., 2017): *HSP101* (*AT1G74310*), *ROF1* (*AT3G25230*) and *GOLS1* (*AT2G47180*). These were tracked during 60 min of high light (HL) followed by either 30 min of recovery (REC), or an additional 30 min of HL (Figure S1). In general, changes in pre‐mRNA mirrored those in mRNA during HL and REC. However, during REC, both *HSP101* and *ROF1* pre‐mRNA levels dropped below pre‐stress levels, suggestive of decreased transcription. During the same period, *ROF1* and *HSP101* mRNA levels remained elevated over pre‐stress levels by approximately fourfold and eightfold, respectively. In contrast, *GOLS1* pre‐mRNA levels followed mRNA levels more closely during REC, and never dropped below pre‐stress levels. Nonetheless, in the case of continued HL stress, pre‐mRNA levels of all three genes remained elevated compared with pre‐stress and REC. This suggests that locus‐specific transcriptional changes contribute towards RRGD.

### Syringe infiltration of cordycepin effectively inhibits transcription in mature leaves

We next sought to quantify *in planta* transcript half‐lives in mature soil‐grown plants during stress and recovery. Owing to the limitations of existing methods, half‐life measurements are typically performed on juvenile seedlings, grown on nutrient‐rich media, or with cell culture (Chantarachot et al., 2020; Narsai et al., 2007; Sorenson et al., 2018; Szabo et al., 2020). While these systems allow for the effective administration of transcriptional inhibitors (e.g. cordycepin or actinomycin D) or uridine analogues for pulse labelling, they do not allow for studies of mature soil‐grown plants alongside applications of external stimuli (e.g. abiotic stress). Therefore, it is currently unclear whether existing measurements of RNA half‐lives are reflective of mature soil‐grown plants. To address these limitations, we developed a method of administering cordycepin that allowed for the simultaneous application of stress treatments. Syringe infiltration was considered a viable procedure for administering cordycepin into the cells of mature Arabidopsis leaves, which presented two advantages. First, it allowed for *in situ* treatment of soil‐grown plants during light stress. Second, since the treatment was applied to individual leaves, a mock treatment could be applied to a separate leaf on the same plant as a control. We could also test for systemic effects by comparing these responses to a third untreated leaf.

**Figure 1:**
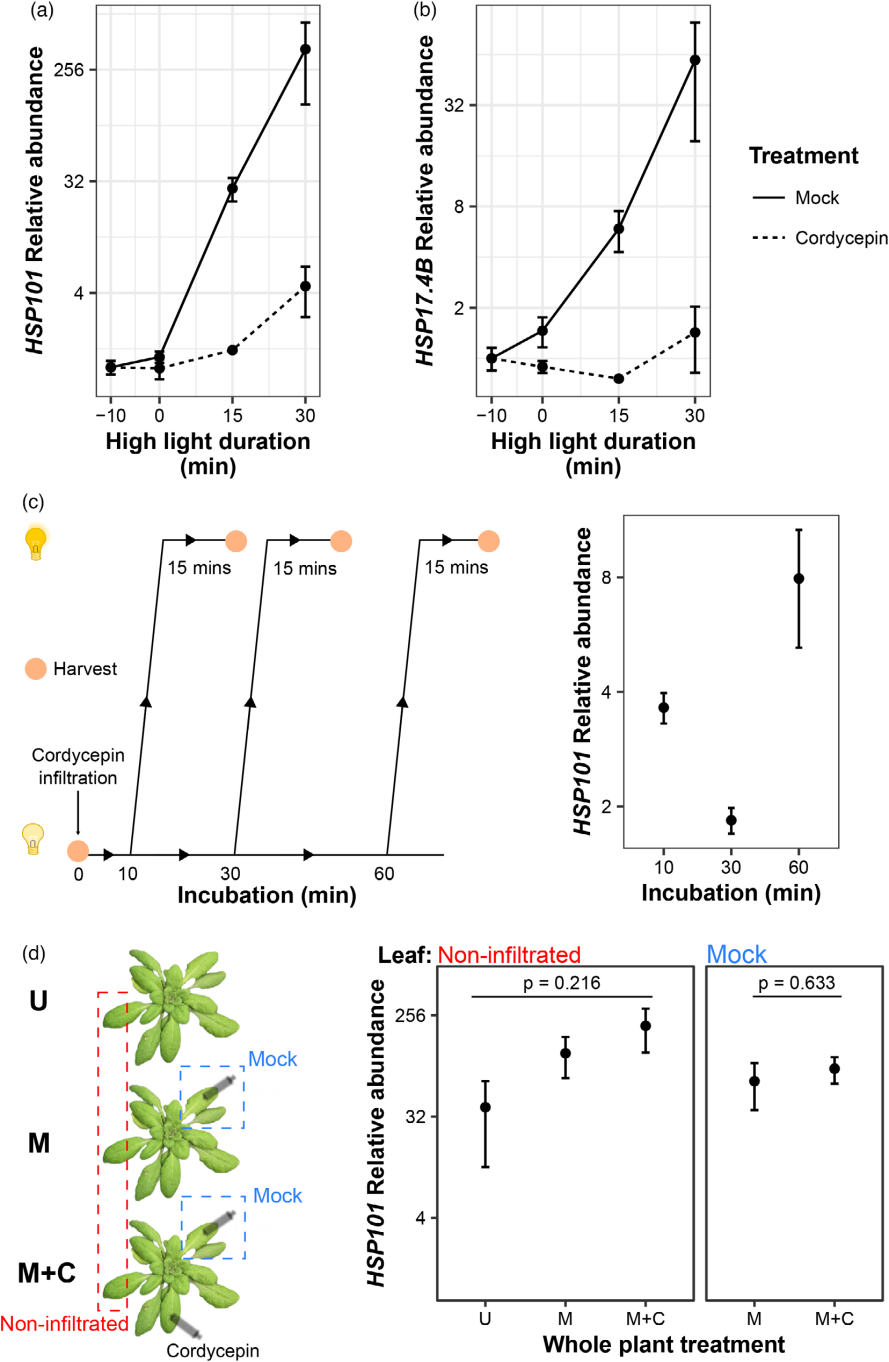
Syringe infiltration of cordycepin inhibits high light‐induced transcription without introducing systemic effects. (a, b) High‐light induction of *HSP101* and *HSP17.4B* was measured over 30 min following infiltration of individual leaves with mock or cordycepin solution. Points denote means, error bars denote standard error of the mean (*n* = 2). (c) Arabidopsis plants were syringe infiltrated with cordycepin and then incubated for 10, 30, and 60 min before exposure to 15 min of high light (*n* = 3). (d) Arabidopsis plants were subject to three different infiltration regimes: untreated (U), mock infiltration (M), or mock and cordycepin infiltration (M + C), on independent leaves. Treatment was followed by 15 min of light stress prior to sampling untreated and mock‐infiltrated leaves. Points denote means; error bars denote standard error of the mean (*n* = 3).

We first evaluated the ability to inhibit transcription by using syringe infiltration into individual leaves before exposure to light stress and measurement of light‐responsive gene induction by qRT‐PCR (Figure 1). While strong induction of *HSP101* and *HSP17.4B* was observed when infiltrated with a mock buffer, this was dramatically attenuated when infiltrated with cordycepin for 10 min (Figure 1a,b). For example, *HSP101*, which was upregulated 375‐fold after 30 min of high light in mock‐treated leaves, was only elevated 4.5‐fold in the presence of cordycepin. This equated to a 98.8% attenuation of the response. A similar block of induction was observed for *HSP17.4B*. We next examined how long cordycepin remained effective after infiltration into mature plant leaves, an important consideration for RNA half‐life measurements. Leaves were infiltrated with cordycepin for 10, 30, or 60 min under unstressed (US) conditions, before exposure to 15 min of stress, with efficacy measured via induction of *HSP101* (Figure 1c). Relative to the 32‐fold induction in the absence of cordycepin, the highest induction (or lowest attenuation) achieved was approximately eightfold when light stress was applied after 60 min of cordycepin incubation, equating to 75% inhibition. However, substantial transcriptional inhibition was observed after 10 (3.8‐fold induction, 88% inhibition) and 30 min (1.5‐fold induction, 95% inhibition) of incubation. It was uncertain whether the application of cordycepin to one leaf could cause systemic effects, which would affect the ability to mock infiltrate a separate leaf on the same plant as an internal control. We tested this by comparing gene induction in response to 15‐min light stress in non‐infiltrated and mock‐treated leaves of separate plants subject to different treatments (Figure 1d). These included untreated (U), mock infiltration (M) or mock infiltration on one leaf and cordycepin infiltration on a second leaf (M + C) on the same plant. Additionally, a non‐infiltrated leaf was also taken from each plant for comparison. For non‐infiltrated leaves, no significant difference in *HSP101* induction was observed between treatment regimes (anova, *P* = 0.216), nor for mock‐treated leaves (unpaired Student's *t*‐test, *P* = 0.633). But in both cases, incremental increases in mean expression were observed as the number of infiltrated leaves per plant increased. While not statistically significant, this suggested that the infiltration itself could stimulate stress‐responsive genes. Critically, it was clear that transcriptional inhibition following application of cordycepin did not impair the induction of genes profiled in distal untreated or mock‐infiltrated leaves (compare to Figure 1c – 10 min). Taken together, we concluded that syringe infiltration presented an effective means of cordycepin administration to inhibit transcription in Arabidopsis leaves.

### Transcripts with reduced stability in mature leaves encode proteins involved in RNA processing and post‐transcriptional regulation

**Figure 2:**
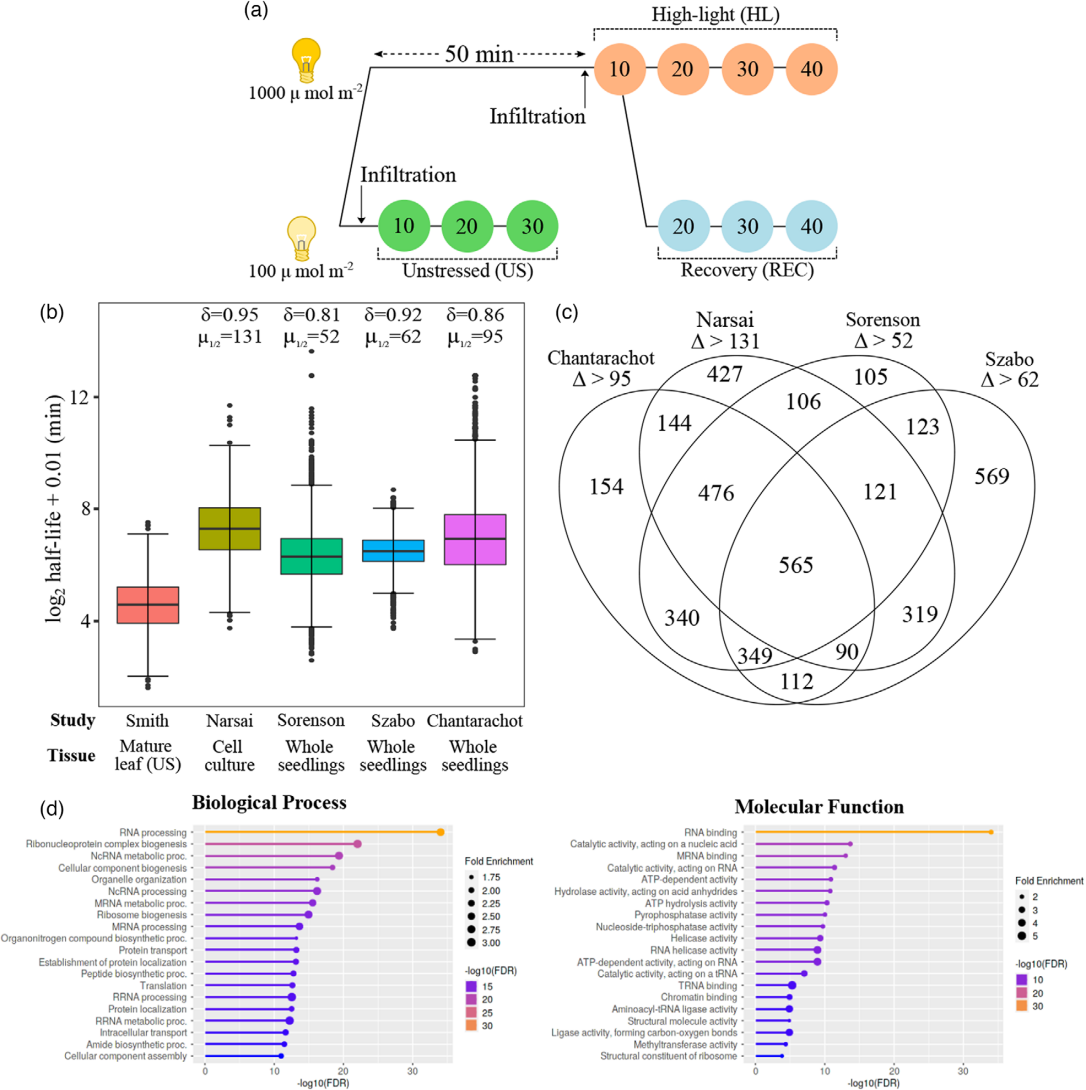
Sampling strategy to determine mRNA half‐lives in Arabidopsis leaves. (a) Unstressed (US) plants were harvested in standard growth conditions (US, 100 μmol m^−2^ sec^−1^) at three time points in 10‐min intervals post‐infiltration. High‐light (HL) and recovery (REC) plants were exposed to 60 min of light stress (HL, 1000 μmol m^−2^ sec^−1^), with infiltration occurring at 50 min. Following initial harvesting at 60 min of HL, plants were either kept under HL or moved for REC (100 μmol m^−2^ sec^−1^) for three harvesting points at 10‐min intervals. (b) Boxplots depicting log_2_ half‐life determined for 4497 genes under US conditions here, and their corresponding values from other studies. Box denotes median and interquartile range, bars denote 1.5× interquartile range, and points denote outliers. *δ* denotes Cliff's delta (the likelihood of observing a difference between groups) compared with this study. *μ* denotes the paired median difference compared with this study. (c) Venn diagrams representing the number of genes measured, in this study, with a reduced half‐life greater than the median difference for each independent dataset assessed. (d) Top enriched gene ontology terms for products of transcripts with reduced half‐life in mature Arabidopsis leaves.

With a functional cordycepin assay established for use under high light, a mRNA‐sequencing time course was performed to estimate RNA decay under US, HL, and REC conditions (Figure 2). In each instance, infiltration of the mock or cordycepin solution was followed by harvesting at 10‐min intervals (Figure 2a). Based on our data, our first sample was taken after 10 min of incubation to ensure effective transcriptional inhibition was achieved prior to sampling (Figure 1c). Since we did not sample immediately upon cordycepin treatment (i.e. *t*_0_), we measured the extent to which this would impact RNA half‐life estimation. Examining a previous cordycepin time course in Col‐0 (therein termed *sov*) (Sorenson et al., 2018), we performed log‐linear regression on normalised and scaled transcript abundances to calculate half‐lives when *t*_0_ was included or omitted (Figure S2a). The omission of *t*_0_ reduced the number of genes able to be statistically modelled (*P* < 0.05) from 16 659 to 16 032. However, for the 15 765 genes modelled in both scenarios (with and without *t*_0_), a high correlation in half‐life was observed (Pearson's *r* = 0.99, *R* ^2^ = 0.98). Similar results were observed when both *t*_0_ and *t*_7.5_ were omitted (13 366 genes, Pearson's *r* = 0.97, *R* ^2^ = 0.94). This analysis suggests that the omission of *t*_0_ will reduce the number of genes that can be modelled across each of our time courses. Critically, however, for those genes that are captured, the half‐life estimates will be negligibly affected. Therefore, this sampling approach was considered suitable for our purpose of quantifying changes in RNA half‐life under different conditions.

The transcriptomes of mock‐ and cordycepin‐infiltrated samples were analysed using mRNA sequencing. Multidimensional scaling plots highlighted the similarity between replicates, confirming the reproducibility of syringe infiltration and stress treatments (Figure S2b). The primary source of sample differentiation was infiltration, which was most pronounced after 30 or 40 min. The second source of differentiation was exposure to stress, with HL and REC co‐clustering separately from US samples. To examine whether syringe infiltration of cordycepin led to widespread transcriptional inhibition, differential gene expression was assessed between the first and last time points following cordycepin and mock infiltration (Figure S2c; Table S4). For mock‐treated samples, no particular bias towards up‐ or downregulation was observed; by contrast, strong downregulation was observed after cordycepin treatment in each condition. For example, during recovery 5178 genes were downregulated following cordycepin infiltration, while just 584 genes were upregulated. The upregulation likely represents the preservation of relatively stable transcripts that become comparatively more abundant as the RNA pool shrinks, resulting in the appearance of increased expression. We utilised decay factor normalisation to reduce this artefact (Sorenson et al., 2018); however, some transcripts still showed a slight level of induction. Nonetheless, the induction of genes by the mock treatment was largely abolished in each condition (US: 95%, HL: 75%, REC: 74%) and the magnitude of downregulation was greater following cordycepin treatment compared with mock. Taken together, these data confirm that widespread transcriptional inhibition was achieved in mature Arabidopsis leaves using syringe infiltration of cordycepin.

We next calculated transcript half‐lives across the transcriptome in unstressed Arabidopsis. Current measurements in Arabidopsis are determined using tissue culture with actinomycin D treatment (Narsai et al., 2007), or nutrient‐rich media‐grown juvenile seedlings using uridine analogues (Szabo et al., 2020) or cordycepin treatment (Chantarachot et al., 2020; Sorenson et al., 2018). We compared measurements with these systems to observations in mature soil‐grown Arabidopsis leaves. The decay constant (*k*_d_) was determined using log‐linear regression of normalised fractional decreases of mRNA abundance, as a function of time, in cordycepin‐infiltrated samples. This allows for the calculation of transcript‐specific half‐lives that were used for comparisons. From the unstressed samples, half‐lives could be computed for 6711 genes based on statistical appropriateness of the fitted regression (Table S5, *P* < 0.05). Half‐lives were extracted from the aforementioned studies, for which 4497 genes could be directly compared with our observations (Figure S2d). This direct comparison hinted at shorter half‐lives in mature leaf tissue compared with prior studies in seedlings and cell culture. Indeed, the transcript half‐lives observed in this study had a median of 24.1 min, which is substantially lower than the prior studies with median half‐lives between 79.2 and 157.6 min (Figure 2b). This was further supported by computing bootstrapped effect size parameters (mean difference, *θ*; median difference, *μ*_1/2_; Cliff's delta, *δ*) (Table 1). In particular, the bootstrapped median difference in transcript half‐lives between datasets varied between 52 and 131 min, with corresponding Cliff's delta in the range of 0.81–0.95 (where a positive value represents the likelihood of observing a larger value). Larger values were observed when computing mean differences between datasets, likely caused by a small number of transcripts that displayed very large half‐lives in some datasets.

**Table 1:** Effect size parameters for differences in transcript half‐life between this study and other datasets

Comparison study	Mean difference (*θ*)	Median difference (*μ*_1/2_)	Cliff's delta (*δ*)
Narsai et al. (2007)	180	131	0.946
Sorenson et al. (2018)	94.8	52.4	0.808
Szabo et al. (2020)	70.1	62.1	0.922
Chantarachot et al. (2020)	352	94.8	0.858

Next, we explored which transcripts exhibited reduced stability in mature Arabidopsis leaves and their biological roles. To define transcripts with lower stability, we compared half‐lives obtained here with each published dataset on a gene‐by‐gene basis. Those with lower half‐lives, by a difference greater than the median difference, compared with each dataset were collated (Figure 2c). There were 1601 transcripts with consistently lower stability compared with the existing studies (lower than three out of four prior measurements; Table S6). These had enrichments of gene ontology terms relating to post‐transcriptional and translational processes (Figure 2d), such as RNA processing, ribosome biogenesis, RNA binding and RNA catalysis (including pyrophosphatase and nucleoside triphosphatase activity). Therefore, transcripts encoding proteins involved in RNA processing, splicing and translation exhibit reduced stability in mature Arabidopsis leaves.

### Changes in transcript stability during light stress and recovery

**Figure 3:**
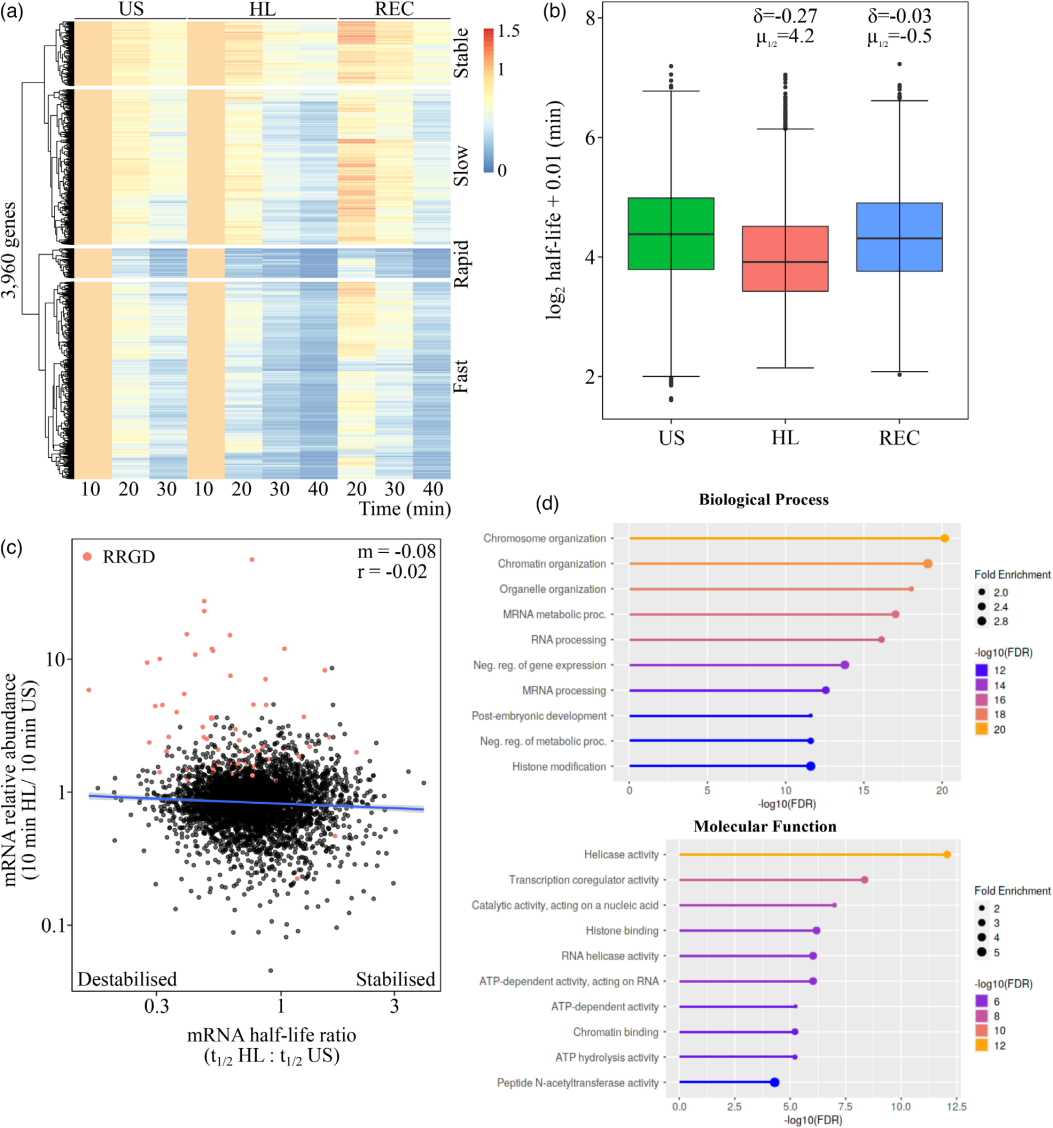
Transcript destabilisation occurs during light stress in Arabidopsis. (a) Heatmap with one‐dimensional hierarchical clustering depicting relative abundance of 3960 genes in cordycepin‐infiltrated samples under unstressed (US), high‐light (HL) and recovery (REC) conditions. (b) Boxplots depicting log_2_ half‐life determined for 3960 genes under US, HL and REC conditions. Box denotes median and interquartile range, bars denote 1.5 × interquartile range, and points denote outliers. *δ* denotes Cliff's delta, *μ*_1/2_ denotes median difference. (c) Scatter plot presenting the fold change in mRNA abundance (10 min HL/10 min US), in mock‐treated samples, versus the half‐life ratio (HL/US) for 3960 modelled transcripts. Points represent individual genes, line denotes fitted linear model, ‘*m*’ denotes regression coefficient, and ‘*r*’ denotes Pearson's correlation coefficient. (d) Top enriched gene ontology terms for products of transcripts destabilised under HL.

Profiling transcript abundance in the presence of transcriptional inhibitors under US, HL and REC allowed us to explore whether mRNA stability demonstrated conditionality (Figure 3). To do this, we first computed half‐lives for 3960 genes that could be statistically modelled under all conditions (*P* < 0.05, Figure 3a; Table S7). Hierarchical clustering of these transcripts revealed four broad patterns of decay, which we define as stable, slow, fast and rapid. These distinct mRNA decay patterns are also visualised for two representative genes in each category across each condition (Figure S3). We then explored whether any functional terms were enriched across these categories. Within the stable category, there were enrichments such as RNA processing (FDR = 6.6 × 10^−20^) and splicing (FDR = 9.6 × 10^−9^), intracellular transport (FDR = 1.9 × 10^−17^) and cellular localisation (FDR = 4.8 × 10^−12^), and organonitrogen compound biosynthesis (FDR = 4.9 × 10^−11^). Slow decay genes exhibited enrichments for organelle organisation (FDR = 7 × 10^−20^), chromosome organisation (FDR = 2.7 × 10^−13^), post‐embryonic development (FDR = 2 × 10^−8^) and RNA processing (FDR = 3 × 10^−22^). Genes within the fast category were enriched for chromosome (FDR = 4.4 × 10^−14^) and chromatin organisation (FDR = 4.4 × 10^−14^), protein modification (FDR = 2.1 × 10^−14^), transcription (FDR = 2.3 × 10^−10^) and negative regulators of gene expression (FDR = 1.3 × 10^−10^). Lastly, rapid decay genes encoded proteins involved in hormone signalling (FDR = 9 × 10^−6^), including responses to gibberellin (FDR = 2.5 × 10^−3^) and ethylene (FDR = 4.1 × 10^−3^), and protein modifications (FDR = 1 × 10^−4^), such as ubiquitination (FDR = 5.6 × 10^−5^).

Transcript abundance appeared to decline faster under HL, suggesting lower stability during stress. Curiously, there was a transient increase in abundance after 10 min of REC, particularly in stable and slow‐decaying transcripts. It is unclear whether this reflects residual transcription or is the result of these transcripts occupying a greater portion of the RNA pool as others are degraded. A comparison of half‐lives between conditions suggested that transcripts were less stable under HL (Med *t*_1/2_ = 15.1 min) compared with US (Med *t*_1/2_ = 20.8 min) and REC (Med *t*_1/2_ = 19.8 min, Figure 3b). We utilised estimation statistics to empirically test whether mRNA stability was altered between conditions in a transcript‐specific manner. While the bootstrapped mean difference was only −0.67 min (*δ* = −0.03) between US and REC, a difference of −5.69 min (*δ* = −0.27) was observed under HL (Table 2). This suggests that transcripts are destabilised under HL, but are then re‐stabilised to pre‐stress levels during REC.

**Table 2:** Effect sizes parameters for differences in transcript half‐life between conditions

Contrast	Mean difference (*θ*)	Median difference (*μ*_1/2_)	Cliff's delta (*δ*)
HL–US	−5.69	−4.21	−0.268
REC–US	−0.668	−0.509	−0.0326

HL, high light; REC, recovery; US, unstressed.

We then tested whether our RNA half‐life analysis was biased towards low‐abundance transcripts, which may be prone to destabilisation. However, it was clear that the 3960 transcripts captured in our analysis were of widely varying abundance (Figure S4a). Next, comparing the half‐life ratio under HL (*t*_1/2_ HL:*t*_1/2_ US) with the fold change in mRNA abundance revealed a negligible relationship (Figure 3c). However, there appeared to be a subset of highly induced transcripts that were destabilised and another portion of transcripts with decreased abundance and increased stability. Of the 388 RRGD loci defined previously (Crisp et al., 2017), 70 were captured in this RNA half‐life analysis. As expected, these were predominantly increasing in abundance (64/70 = 91.4%) when contrasted between HL and US in mock‐treated leaves. Indeed, these 64 genes comprised the majority of the most highly upregulated genes by HL. Within this upregulated subset, there was a clear bias towards destabilisation (55/64 = 85.9%), which suggests a coupling between induction (or increased abundance) and destabilisation. Conversely, these RRGD transcripts were largely downregulated and stabilised in the transition from HL to REC (Figure S4b).

We define 1980 HL destabilised transcripts based on observing a reduction in half‐life greater in magnitude than the median difference across transcripts between HL and US (i.e. 4.21 min, Table S8). These genes were enriched for those encoding proteins involved in transcriptional regulation, such as chromosome and chromatin organisation, and transcription coregulator, histone binding and helicase activities (Figure 3d). Paired with this were terms related to the (negative) regulation of metabolic processes and post‐embryonic development. Once more, terms related to post‐transcriptional regulation were observed, albeit to a lesser extent, with enrichments in RNA helicase activity, mRNA metabolism and RNA processing. Therefore, light stress appears to induce the destabilisation of transcripts involved in both transcriptional and post‐transcriptional regulation, as well as the transition towards reproductive growth. Since it appeared that destabilisation of transcripts occurred under HL, we investigated the timing of this destabilisation (Figure S4). To test this, plants were exposed to high light for 60 min, with subsets of plants infiltrated with mock or cordycepin solution at 5, 20, and 50 min. Samples were then harvested after 10, 20, and 30 min to measure decay rates using qRT‐PCR of *AT3G14200*, which exhibited HL‐induced destabilisation based on the mRNA sequencing (Figure S5a). Interestingly, plants infiltrated at 5 min of HL exhibited an increase in *AT3G14200* in the 10 min following infiltration, which may be attributed to residual transcription and RNA processing while the cordycepin took effect on newly synthesising transcripts (Penman et al., 1970). However, transcript levels did decline from 20 to 40 min. In both the 20‐ and 50‐min treatment groups, steeper and more consistent declines were observed in the 30 min following infiltration. To quantify these differences, RNA half‐life was modelled using the period 10 to 30 min post‐infiltration. The half‐life of *AT3G14200* after 5 min of HL was estimated at 35.58 min, but this model was not significant (*P*_adj_ = 0.149). By contrast, the half‐lives following infiltration at 20 and 50 min of HL were estimated at 19.91 and 16.45 min, respectively (*P*_adj_ = 0.00027). This indicated that *AT3G14200* was more stable during the initial period of high light stress and subsequently destabilised.

The finding that *AT3G14200* is destabilised based on the duration of stress suggests that RRGD, typically observed after 60 min, may not occur with shorter durations of HL. To test this, plants were exposed to 10, 30, 60, and 120 min of HL before being moved to REC (Figure S5b–d). Quantification of relative changes in RNA abundance from canonical RRGD loci: *HSP101*, *ROF1* and *GOLS1* using qRT‐PCR revealed that 10 min of HL did not cause recovery‐induced downregulation. By contrast, clear declines in transcript levels were observed when the plants were moved to REC after 30 and 60 min of HL, while a small increase was observed followed by a decline after 120 min. Gene expression patterns seen in *GOLS1* were similar, though declines in transcript levels were observed in the last 10 min of REC after each HL length. Estimations of RNA half‐lives were modelled for each REC period, however, following adjustments for multiple comparisons, no model was significant (*P* < 0.05). Nonetheless, they did indicate the same general trend, with the most notable downregulation being observed following 60 min of HL. This reinforced the cordycepin data obtained for *AT3G14200* and indicated that RNA from RRGD loci remain comparatively stable in the early stages of HL before being destabilised.

### Translational regulation occurs during late stress and early recovery

**Figure 4:**
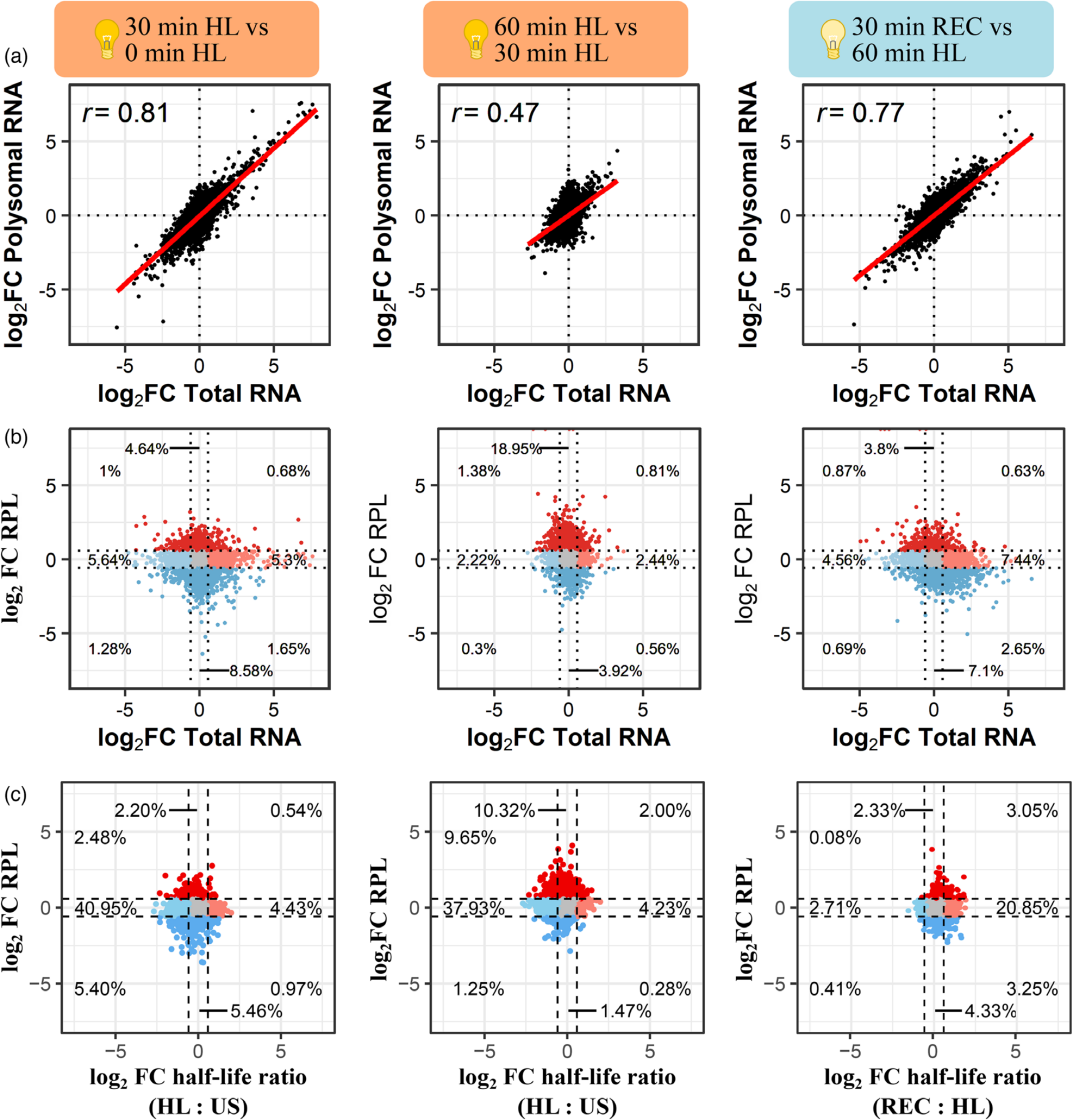
Translational regulation during light stress and recovery in Arabidopsis. (a) Scatter plots comparing gene‐specific fold changes in total and polysome‐bound mRNA abundance between time points. (b) Scatter plots comparing fold changes in total mRNA and relative polysome loading (RPL = polysomal mRNA/total mRNA) between time points. (c) Scatter plots comparing fold changes in RPL and half‐life ratios between time points, relating transcript half‐life to their abundance in the polysome‐bound mRNA fraction and RPL. Colouring indicates fold change greater (red) or less (blue) than 1.5 for either total mRNA or RPL. Numbers denote proportion of transcripts detected in each section. Points represent individual transcripts, line denotes fitted linear model, and ‘*r*’ denotes Pearson's correlation coefficient.

To investigate the interplay between transcript stability and translation during HL and REC, we sequenced polyribosome (polysome)‐associated mRNAs, which represent transcripts involved in translation (Lecampion et al., 2016). This was done after 30 and 60 min of HL and after 7.5, 15, and 30 min of REC (Tables S9–S11). To achieve a reproducible recovery of polysomes from plant material, we used an extraction buffer with a low concentration of monovalent salts (80 mm K^+^) and high concentrations of translational inhibitors (150 μg ml^−1^ cycloheximide and 150 μg ml^−1^ chloramphenicol) in order to robustly stabilise elongating ribosomes, similar to the conditions suggested previously (Hsu et al., 2016). During polysome fractionation, we generated in‐line UV‐absorbance profiles to locate monosomal and polysomal fractions and control for any shifts in the global translational landscape (Figure S6a). Consistent with Hsu et al. (2016), the profiles exhibited a high monosome‐to‐polysome ratio. This is likely the combined result from using a modified extraction buffer and the nature of our material, since older Arabidopsis leaves have reduced polysome content (Carpentier et al., 2020). Nevertheless, the polysome profiles were highly reproducible and, therefore, were considered reliable for making comparisons in polysome‐associated RNA. The RNA quantities recovered from each fraction were also highly consistent between the samples, indicating little alteration in the overall transcript amount associated with polysomes (Figure S6b). While no major disruption was observed, individual transcripts may still undergo substantial translational regulation. To examine this, we sequenced the total (lysate before ultracentrifugation) and polysome‐associated mRNA populations, then calculated relative transcript abundances at the specified time points on a per‐gene basis (Figure 4). To further validate the quality of our material and reproducibility with the published observations, we compared polysome‐associated mRNA sequencing performed here on unstressed plants (0 min HL) with those reported previously on 25‐day‐old Col‐0 (Carpentier et al., 2020). Indeed, gene‐level abundances were highly correlated between both RNA‐sequencing datasets (Pearson's *r* = 0.84), ensuring the robustness of the downstream analyses (Figure S6c). Multidimensional scaling was used to assess the similarity in polysome‐associated and total mRNA quantification across samples, which confirmed reproducibility between biological replicates (Figure S6d). Intriguingly, changes in total and polysome‐associated mRNA were highly correlated (Pearson's *r* = 0.81) in the early stages of stress (0–30 min) indicating loading of transcripts onto polysomes proportional to changes in transcript abundance (Figure 4a). But, during the later period of stress (30–60 min), the two populations were substantially less correlated (Pearson's *r* = 0.47). At early REC time points, the changes between the two populations were still weakly linked (Pearson's *r* = 0.25, Figure S6d). However, this correlation was gradually restored over 30 min REC (Figure 4a, Pearson's *r* = 0.77). This decoupling of total and polysome‐associated mRNA abundance during late stress and early recovery is indicative of an onset of translational regulation. Notably, this suggests that changes in translation are occurring independently of a concomitant change in total transcript abundance, and that the translational changes are invoked in a transcript‐specific manner.

As ribosomes are recruited onto transcripts across the total cellular pool, it can be informative to examine the ratio between polysome‐associated and total mRNA in a transcript‐specific manner. While this would not be a direct reflection of the specific yield of translation, which includes rates of ribosomal attachment, progression and dissociation over mRNA and not only ribosomal loading, the proportion of the polysome‐associated mRNA is a solid indicator of mRNA translational propensity. We refer to this ratio as relative polysome loading (RPL), where a value of one indicates that polysome‐associated and total mRNA occur at proportionally the same relative abundance. It is important to note that changes in RPL can occur via differential recruitment of ribosomes to transcripts (numerator) or changes in total transcript abundance (denominator). Notably, RPL can increase even if levels of polysome‐associated mRNA decrease, provided this occurs at a slower rate than a reduction in total mRNA abundance. Therefore, comparing changes between RPL and total mRNA can be used to more easily identify shifts that occur in only one RNA population. This comparison was performed for three periods: early HL (0–30 min), late HL (30–60 min) and REC (Figure 4b). On a broad scale, clear differences could be observed between the three periods although a striking proportion of transcripts showed changes in RPL without changes in total mRNA abundance (early: 13.22%, late: 22.87%, REC: 10.9%). In each period, these changes were enriched for regulation in opposing directions with early HL and REC being biased towards reduced RPL, whereas late HL showed a striking enrichment of genes with increased RPL. Such results indicate a bias towards translational repression at the onset of stress, followed by increased propensity to translate. Furthermore, the 858 transcripts with increased RPL during late HL (Table S12) were enriched for functions related to transcription (e.g. transcription regulator activity, FDR = 8.7 × 10^−5^), macromolecule methylation (e.g. RNA methylation, FDR = 6.3 × 10^−5^), RNA processing (FDR = 2.1 × 10^−8^), splicing (FDR = 1.2 × 10^−4^) and organelle organisation (FDR = 1.2 × 10^−4^). Notable too were the changes in the proportion of transcripts showing differences in RPL relative to total mRNA, reflecting disproportionate changes in polysome association compared with transcript abundance (Figure 4b). During early stress, 20.8% and 19.2% of transcripts exhibited changes in RPL and total mRNA, respectively, a 1.1:1 ratio. During late stress, 25.9% and 7.7% of transcripts had changed RPL and total mRNA, respectively, a 3.4:1 ratio (threefold increase). In recovery, this returned to 15.7% and 16.8% changes in RPL and total mRNA, respectively, reflecting a 0.9:1 ratio. Overall, these results highlight the extent of translational regulation during light stress and recovery, with later stress (30–60 min of HL) being a dynamic period with increased ribosomal loading of many transcripts independently of changes in total mRNA abundance.

Since transcript stability may influence translation, we examined the relationship between half‐life and RPL during the same early HL, late HL and REC periods. We hypothesised that destabilised transcripts might show reduced polysomal association. To test this, we related the fold change in RPL, measured for each period, to the half‐life ratio (i.e. the change in stability) observed under HL and REC for 3905 genes detected in each dataset (Figure 4c). Unexpectedly, there was no clear relationship between changes in RPL and mRNA stability. Under both periods of HL, there was a large proportion of transcripts that were destabilised yet had unchanged RPL, indicating that the majority of destabilised transcripts retained similar levels of polysome recruitment. Conversely, during recovery there is a clear increase in the stability of many transcripts yet the majority of these also showed no difference in RPL. Our results suggest that changes in mRNA stability are not strongly linked with changes in polysome association or dissociation, and that the cytoplasmic dynamics of mRNA stability and translation have complex, non‐uniform relationships during plant stress and recovery.

### Dynamics of transcripts from RRGD genes can impact their translation

We previously defined RRGD genes as those upregulated at least threefold in their mRNA abundance after 30 min of HL. These were categorised based on their expression profiles: category 1 genes remained upregulated through HL and REC, category 2 genes were downregulated during REC, and category 3 genes were downregulated after 30 min of HL (Crisp et al., 2017). However, it was unclear whether these dynamics had functional consequences. Therefore, we investigated whether the mRNA dynamics displayed by RRGD genes impacted translation. Examination of polysome‐associated transcripts found that they exhibited an expression profile concordant with that observed in total mRNA (Figure S7). This suggests that, contrary to the decoupling observed on a global scale, the free and polysome‐associated mRNA populations were tightly linked for RRGD genes. That is, changes in mRNA abundance of an RRGD gene generally led to changes of corresponding direction in that mRNAs translation. This observation indicates that the phenomenon of RRGD is likely to have tangible cellular consequences by impacting protein production.

**Figure 5:**
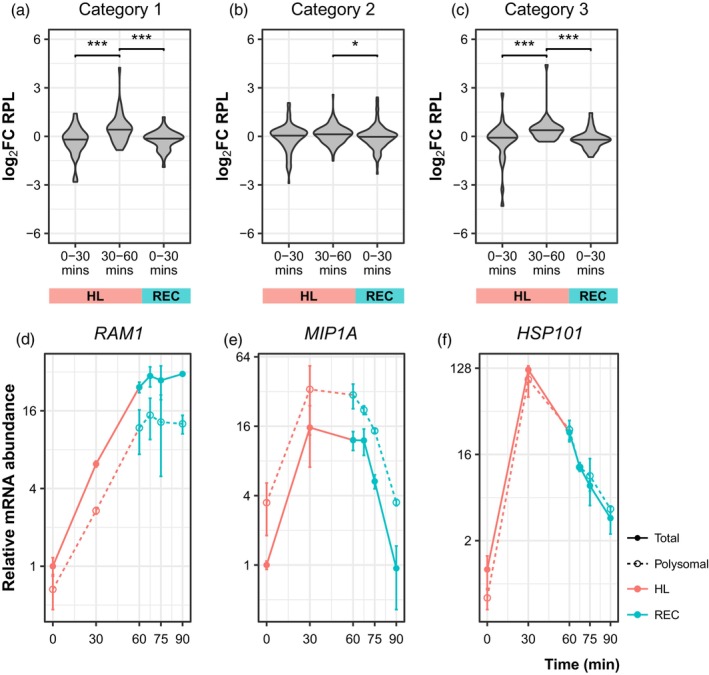
Rapid recovery gene downregulation (RRGD) can impact translation. (a–c) RRGD genes were defined as those upregulated at least three‐fold after 30 min of high light (HL). These were categorised based on their expression profiles: category 1 genes remain upregulated through HL and recovery (REC), category 2 genes are downregulated during REC, and category 3 genes are downregulated after 30 min of HL. Changes in relative polysome loading (RPL) for genes across these categories were compared across the three 30‐min time periods. Median foldchange was compared using Wilcoxon rank‐sum test, *P < 0.05, ***P < 0.001. (d–f) Examples of gene expression profiles in each category. Points denote mean; error bars denote SE (*n* = 2).

Increased indices of co‐translational decay were also observed in RRGD genes, suggesting that co‐translational decay is involved in the removal of transcripts during recovery (Crisp et al., 2017). Therefore, we hypothesised that RRGD genes may exhibit reduced RPL, as their polysome‐associated transcripts are degraded at a higher rate than free unbound transcripts. To test this, changes in RPL were compared between each category of RRGD gene across the time course (Figure 5). Genes in categories 1 and 3 tended to have an increase in RPL during 30–60 min of HL (Figure 5a–c). This was particularly notable for category 3 genes that exhibited reduced total mRNA abundance (Figure 5d–f). However, all categories of RRGD genes exhibited declines in RPL during REC, indicating either ribosomal unloading via translational control (e.g. category 1) or dynamic degradation (e.g. categories 2 and 3) of polysome‐associated transcripts.

## DISCUSSION

**Figure 6:**
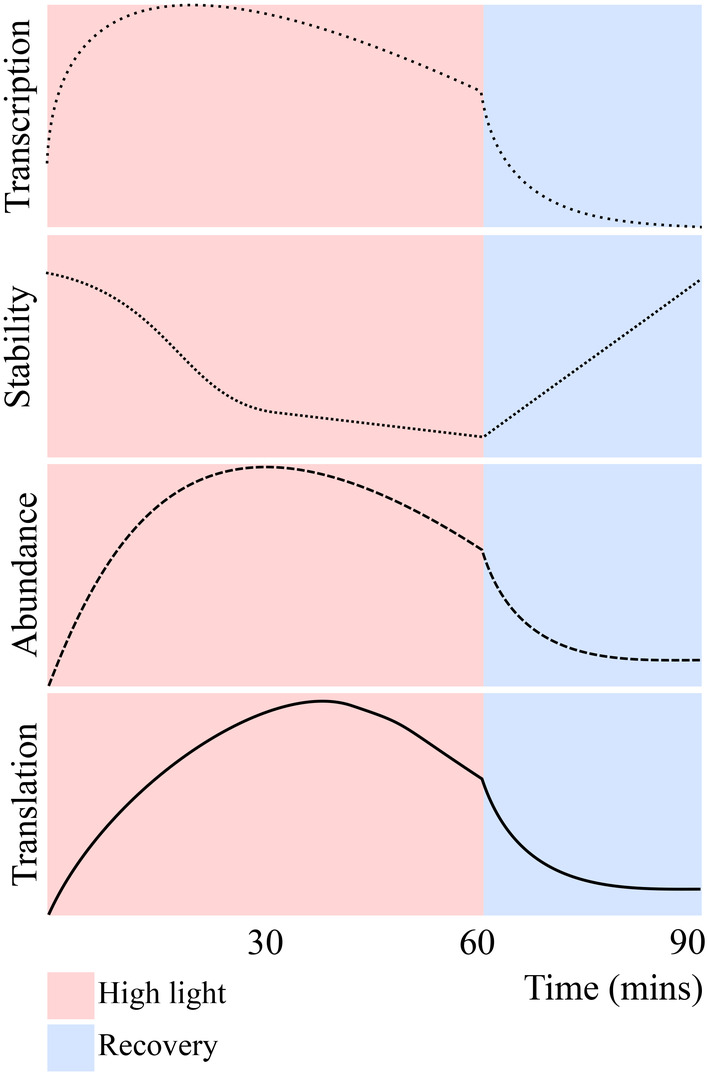
Stress‐induced transcript destabilisation facilitates rapid recovery gene downregulation (RRGD). A proposed model of RRGD transcript dynamics during high (HL) light and recovery (REC). Transcription of RRGD genes is induced by HL, which leads to increased total mRNA abundance. This is paired with transcript destabilisation, which, when coupled with the cessation of transcription in recovery, we hypothesise to be causing the rapid decrease in transcript levels. Critically, we find evidence that these rapid changes in overall mRNA abundance can impact the propensity for these mRNAs to be translated through changes in polysomal loading.

To change gene expression during stress and recovery, plants can alter transcription, mRNA stability and/or translation. Here, we characterise changes during a highly dynamic period for mRNA abundance for each of these processes under light stress and recovery (Crisp et al., 2017). We focus on the extent to which mRNA stability was modulated to control RNA abundance, and the subsequent impact on translation. Light‐induced changes in transcription were indirectly inferred from changes in pre‐mRNA levels for *HSP101*, *ROF1,* and *GOLS1*, all of which showed decreases upon perception of recovery. Meanwhile, mRNA destabilisation occurred under light stress followed by its stabilisation in recovery. Based on detailed expression profiling of *AT3G14200*, this destabilisation appears to occur after 30 min of high light, at which point peak expression is reached. Similarly, recovery‐induced downregulation was not observed for *HSP101*, *ROF1,* and *GOLS1* in plants exposed to 5 min of stress, suggesting that destabilisation is dependent on the duration of exposure. Furthermore, we found that light‐induced transcripts showed concordant changes in polysome loading and total mRNA levels. Based on our findings, we propose a model of mRNA dynamics during light stress and recovery in Arabidopsis (Figure 6). Onset of stress is associated with a burst of light‐induced transcription, which occurs for 30 min. Following this, mRNA stability decreases and transcription slows, leading to slower accumulation in overall abundance. Yet, preferential ribosome association of specific transcripts, encoding light‐induced and regulatory proteins (e.g. transcription and RNA processing), continues to take place during late stress. Upon recovery, the reduction in pre‐mRNA levels suggests that transcription of stress‐induced mRNAs largely ceases. Due to the prior transcript destabilisation, a rapid downregulation in total and polysome‐associated mRNAs occur causing a resetting of the transcriptome to near pre‐stress levels. In this way, changes in mRNA stability act as a precursor to RRGD, maximising the impact of the transcriptional shut‐off by accelerating transcript turnover during this period.

### Dynamic mRNA stability during plant development and light stress

Half‐lives of mRNA were measured in mature Arabidopsis leaves using a newly developed method, based on syringe infiltration of cordycepin. This allowed us to test for differences in mRNA stability in older plants, since existing measures have only been performed in juvenile seedlings. We observed a substantial decrease in half‐life of the majority of transcripts originating from the genes that could be contrasted with existing datasets (Chantarachot et al., 2020; Narsai et al., 2007; Sorenson et al., 2018; Szabo et al., 2020). Those transcripts exhibiting reduced stability in mature leaves were strongly enriched to encode proteins involved in co‐ or post‐transcriptional processes, including RNA processing and catalysis, RNA binding, ribosome biogenesis and translation (Figure 2d). Contrary to mature soil‐grown leaves, juvenile seedlings (or cultured cells) growing in nutrient‐rich media will likely be undergoing higher rates of cell division (Dean & Leech, 1982; Desvoyes et al., 2006; Donnelly et al., 1999) and chloroplast development (Dean & Leech, 1982; Pogson et al., 2015). When paired with a ready supply of the requisite macromolecules, this may be driving higher rates of protein synthesis and turnover (Ishihara et al., 2015; Li et al., 2017). A reduced stability of such transcripts may be allowing for greater responsivity (i.e. reduced mRNA stability allows for shorter transition times) in post‐transcriptional regulation to tailor protein synthesis proportionally to the cell's requirements and metabolic resources. However, there are many variables altered between independently performed studies that could explain the observed differences. Therefore, assaying RNA half‐lives over developmental time (e.g. differing leaf number) and between tissues (e.g. leaf versus flowers) would be an approach to test the extent to which mRNA stability changes across developmental stages.

We previously hypothesised that mRNA stability was modulated during recovery based on observing a rapid decline in the abundance of light‐induced transcripts (Crisp et al., 2017). The use of syringe infiltration allowed *in situ* application of cordycepin to plants undergoing stress and recovery, allowing us to test this hypothesis. Contrary to expectations, we observed transcript destabilisation under high light followed by stabilisation upon recovery (Figure 3b). Our approach allowed us to measure transcript half‐lives within 30 min of the initial response in contrast to the typical hours. Therefore, we capture highly dynamic responses including those of rapidly destabilised transcripts. However, we also acknowledge that a limitation of our approach was the number of time points that could be sampled, which likely impacted regression fit and thereby limited the number of transcripts that could be reliably modelled. Additional sampling time points could have increased the resolution and allowed more transcripts to be modelled; however, even at this resolution, we were able to model the half‐lives for the majority (60%) of transcripts that decayed significantly upon cordycepin treatment. It is also possible that blocking transcription will influence the rate of RNA decay, since studies in yeast suggest that there is an interaction between factors involved in transcription and decay to coordinate steady‐state RNA levels (Haimovich et al., 2013; Sun et al., 2013). Therefore, an orthogonal method to account for changes in the activity of the decay machinery would be beneficial for future studies. A pronounced spike in relative abundance was observed for a subset of transcripts after 10 min of recovery in the presence of cordycepin (Figure 3a). We previously speculated that this response could represent a recovery‐activated mechanism involving the release of a transcriptional repressor (Crisp et al., 2017). Instead, our results suggest that this spike may reflect residual transcription through some ‘inertia’ of the regulation, based on observing steady pre‐mRNA levels from late stress into recovery (Figure S1), paired with changes in transcript stability. Perhaps, this inertia is a feature of gene expression preventing excessive mRNA and protein throttling, and the associated energy expense, during spurious environmental stimuli. Nonetheless, half‐life measurements over 3960 transcripts revealed destabilisation during light stress (median half‐life decrease = 4.21 min), which appears to facilitate RRGD. We hypothesise that 20 min of stress are required for this destabilisation to develop, based on expression profiling of RRGD loci: *AT3G14200*, *HSP101*, *ROF1,* and *GOLS1* (Figure S4). It remains to be established whether these observations can be generalised across the transcriptome, and whether similar response profiles are invoked for different stressors.

The destabilisation of stress‐induced transcripts has been observed more broadly, including in yeast exposed to oxidative stress, induced by 25 min of hydrogen peroxide (Shalem et al., 2008) and 6–30 min of NaCl (Molin et al., 2009), and Arabidopsis cells undergoing 24 h of cold stress (Chiba et al., 2013). That stress‐responsive genes are destabilised during stress appears counterintuitive. However, this likely confers faster responsivity since unstable transcripts have shorter transition times in reaching new steady‐state levels (Pérez‐Ortín et al., 2007; Ross, 1995). Such an inverse relationship in which unstable transcripts are more quickly induced has been observed in multiple eukaryotes, in a gene‐ and stress‐specific manner (Elkon et al., 2010; Molina‐Navarro et al., 2008). While such a strategy is energetically costly, the benefits are faster responses to both the initial stress and perception of recovery (Shalem et al., 2008). This response is not necessarily global, as transcript‐specific responses are also observed, likely influenced by the type and duration of the stress. For example, 15 min of osmotic shock in yeast led to widespread transcript destabilisation, while a small subset (121 genes, 2.2%) of stress‐induced transcripts were preferentially stabilised (Romero‐Santacreu et al., 2009). In contrast, the stabilisation of stress‐induced transcripts was observed in yeast experiencing a slow enduring stress (40 min of methyl methanesulfonate treatment) (Shalem et al., 2008). Similarly, in Arabidopsis, longer term salt stress (2 weeks) was also associated with the stabilisation of salt‐induced transcripts encoding stress‐response proteins, alongside increased *N* ^6^‐methyladenosine deposition (Anderson et al., 2018; Kramer et al., 2020). This preferential stabilisation likely reflects the activity of mRNA‐binding proteins controlling transcript localisation. For example, SPI functions to relocalise its mRNA targets towards processing bodies for stabilisation during salt stress (Steffens et al., 2015).

### RRGD is facilitated by stress‐dependent mRNA destabilisation and, possibly, transcriptional shut‐off

The rapid decline in RRGD transcript levels in recovery appears to be facilitated by their destabilisation during light stress. Indeed, previously defined RRGD loci that are highly upregulated during light stress were among the most destabilised transcripts (Figure 3c). We hypothesise that transcriptional shut‐off, which should occur once the stimulus for induction is removed, also contributes to this resetting although this was only verified for three RRGD loci: *HSP101*, *ROF1* and *GOLS1*. We estimated transcriptional changes by detecting pre‐mRNA through the use of intronic primers amplifying intron–exon junctions of unspliced mRNA (Zeisel et al., 2011). Such changes are inferred under the assumption that the conversion of pre‐mRNA to mRNA is constant, which in fact could be disrupted by differential splicing, nuclear RNA decay, or nuclear export that may be specifically controlled and timed (Wickramasinghe & Laskey, 2015). Yet, unsurprisingly, changes in pre‐mRNA levels tracked closely with those of mature mRNAs for the genes profiled (Figure S1). However, important differences were observed that likely reflect differential regulation of distinct light‐responsive transcripts. From previous observations, *HSP101* and *ROF1* decline in abundance after 30 min of high light, whereas *GOLS1* declines specifically during recovery (Crisp et al., 2017). For *HSP101* and *ROF1*, pre‐mRNA levels decline to below pre‐stress levels during recovery, while mRNA levels remain comparatively elevated. By contrast, *GOLS1* pre‐mRNA levels more closely resemble those in mRNA and did not decline to pre‐stress levels during recovery. From this, we indirectly infer that there is differential transcriptional repression of light‐responsive transcripts during recovery. However, this requires further investigation with more extensive profiling of transcriptional changes. This will be most practical using genome‐wide measurements of pre‐mRNA levels that may be possible from conventional mRNA sequencing at greater sequence depth (Gray et al., 2014), especially when combined with fractionation of nuclear RNAs, since the short timescales and stress treatments used here make protocols such as base analogue labelling, RNA polymerase II ChIP sequencing and global run‐on sequencing impractical. Notably, however, pre‐mRNA levels began decreasing during the stress period for all three genes assayed, regardless of changes in mRNA. This suggests an initial transcriptional burst, mediated by light‐activated transcription factors, which lasts for approximately 30 min as also observed in mammals and fungi (Cesbron et al., 2015; Li et al., 2018; Molina et al., 2013).

Transcriptome resetting during recovery appears to be programmed by the cell in advance, during light stress, by changes in mRNA stability and transcription. As these are influenced by the duration of stress, it is possible that the recovery response may be altered in the same way. Among studies investigating recovery, it is rare that differing lengths of stress are compared. As demonstrated here, the recovery response is influenced by the duration of stress. Indeed, plants entering recovery after only 10 min of light stress displayed steady transcript abundances (Figure S4). Plants required at least 30 min of light stress before observing the expected rapid decline, which coincides with the mRNA stability data showing transcripts were destabilised by this point. Given that transcription appears to be ongoing at 30 min, this suggests that transcript destabilisation is the primary response for regulation in recovery, with a secondary transcriptional shut‐off occurring during the recovery itself. This coordination of mRNA stability and transcription aligns with observations made in yeast responding to oxidative stress (Molin et al., 2009; Shalem et al., 2008), whereby early‐induced transcripts are destabilised while those repressed are stabilised. This appears to be a mechanism by which cells ‘prepare’ for recovery; upon which the destabilised stress‐induced transcripts are degraded while the stabilised stress‐repressed transcripts accumulate, ultimately resetting the transcriptome to a pre‐stress state.

Impairments in RNA decay enzymes, especially 5′–3′ RNA degradation pathways, can influence plant stress responses (Crisp et al., 2018; Jang et al., 2019; Merret et al., 2013; Nguyen et al., 2015; Soma et al., 2017; Wawer et al., 2018; Xu & Chua, 2012; Yu et al., 2019). However, the mechanism of transcript destabilisation occurring during light stress remains unclear. Indeed, this has been challenging to identify, since RNA decay pathways are complex and redundant, with mutant analyses complicated by both feedback, compensation and lethality of combinatorial mutations. Previous studies profiled a series of combinatorial mutants during recovery; however, the downregulation of stress‐induced genes appeared unperturbed (Armbruster et al., 2019; Crisp et al., 2017). The turnover of mRNA is also regulated by numerous mRNA‐binding proteins, interacting with translation and decay machinery, which requires further exploration (Chantarachot & Bailey‐Serres, 2018). Post‐translational regulation of the activity of RNA helicases targeting stress‐responsive transcripts presents one possible mechanism for stress‐induced mRNA destabilisation (Chantarachot et al., 2020). Another is the involvement of stress‐induced variation in 5′‐terminus NAD^+^‐capping for transcript‐specific changes in stability (Yu et al., 2021).

### Regulation of translation during late light stress and at the onset of recovery

There is an increasing number of reports highlighting the regulation of translation in plants responding to stress, at both global and transcript‐specific levels in conjunction with changes in mRNA turnover and stability (Chantarachot & Bailey‐Serres, 2018). Extending this literature, we find that regulation of translation is dynamic during light stress and recovery. This was evident as transcript‐specific changes in polysome association (Figure S5a,b), as opposed to wholesale re‐organisation elicited by pattern‐triggered immunity (bacterial translation elongation factor EF‐Tu, elf18) (Xu et al., 2017), darkness (Juntawong & Bailey‐Serres, 2012), heat stress (Merret et al., 2017; Zhang et al., 2017) and hypoxia (Mustroph et al., 2009). The modesty in translational response observed here may reflect a stress of lesser intensity, in this case a 10‐fold increase in light irradiance for 60 min. This posits greater complexity to the regulation of translation, at the global and transcript‐specific levels, to be tuned based on environmental stimuli and requirements. This is likely determined through the action of intracellular signalling pathways and their interplay with RNA‐binding proteins and translational machinery, which represents new avenues of investigation. Retrograde signalling has been linked to nuclear RNA processing (Zhao et al., 2020). However, the speed with which translational regulation occurs (e.g. within 7.5 min recovery as measured in Figure S5c) posits direct interaction between chloroplast‐derived signals and cytosolic translation factors or interacting RNA‐binding proteins, many of which are redox‐sensitive (Huang et al., 2019; Moore et al., 2016).

Temporal differences in transcript‐specific translation were clearly observable based on changes in RPL (Figure 4b). During early stress, there was a slight bias towards decreased RPL, indicating translational repression of many genes. This aligns with the observations that stress initially represses translation by perturbing the machinery involved in initiation, scanning and elongation (Bresson et al., 2020; Janapala et al., 2019; Merret et al., 2015; Shalgi et al., 2013; Shirokikh & Preiss, 2018; Woodward & Shirokikh, 2021). On the contrary, we observed a striking increase in RPL during late stress that occurred independently of changes in total mRNA levels. This likely reflects the combination of two phenomena. In the first instance, this may reflect a transient pausing of elongation, without eliciting mRNA degradation, which resumes as cells begin to acclimate to the new conditions, as was observed in yeast (Shalgi et al., 2013). Simultaneously, preferential translation may be occurring for light stress‐associated genes to facilitate an acclimatory response (Chen et al., 2021). The concordance between total and polysome‐associated mRNA levels was also uncoupled rapidly upon transition to recovery, but is eventually re‐established after 30 min (Figure S5c). This reinforces the notion that this is an active period of regulation to establish post‐stress homeostasis to favour growth, as opposed to a passive release of stress defence to return to a pre‐stress condition. Indeed, although physiological and anatomical parameters return to pre‐stressed levels after recovery from non‐lethal stress, differential redox (Brossa et al., 2015), proteomic (Lyon et al., 2016; Schneider et al., 2019; Tamburino et al., 2017) and metabolomic (Lehmann et al., 2012; Wedeking et al., 2018) signatures represent a unique cellular environment, especially during early recovery.

Translation and mRNA stability are coupled by a complex variety of interactions (Roy & Jacobson, 2013). For instance, perturbations to translation, such as ribosome initiation, elongation or unloading, can elicit changes to transcript stability in association with mRNA translation and degradation machinery. For instance, glucose withdrawal rapidly abolished 5′ RNA binding of translation initiation factors within 2 min, leading to translational shutdown without mRNA degradation (Bresson et al., 2020). Conversely, heat shock‐induced progressive loss of 5′ RNA binding of translation initiation factors paired with XRN1‐mediated mRNA degradation within 12 min (Bresson et al., 2020). Given the complexity of these interactions, it is unsurprising that we observed a negligible relationship between changes in RPL and mRNA abundance or stability (Figure 4b,c). For example, many transcripts had altered RPL without significant changes in total mRNA abundance (e.g. 13.22% after 30 min HL, Figure 4b). At the same time, a smaller fraction (6.64% after 30 min HL) of transcripts showed increased or unchanged RPL alongside decreased total mRNA abundance. We hypothesise that these observations represent cases of co‐translational decay. However, further confirmation is required, for example, by assaying RNA cleavage products from the polysome‐associated mRNA pool. Since we previously observed elevated indices of co‐translation decay in RRGD transcripts (Crisp et al., 2017), we expected these to have reduced RPL during late stress or upon recovery. While declines in total mRNA from RRGD genes were generally concordant with declines in polysome‐associated mRNA (Figure S6), RPL tended to increase during late stress (Figure 5). There was a notable decrease in RPL during recovery, especially for category 3 RRGD transcripts, suggesting that polysome‐associated mRNAs were declining faster than free mRNAs. The incorporation of translational inhibitors with this experimental strategy, targeting initiation (Sekiyama et al., 2015) or elongation (Schneider‐Poetsch et al., 2010), may help unpack the fate of light‐induced transcripts when translation is perturbed. Additionally, the use of unbiased translational complex stabilisation, allowing for the capture of rapid responses, and methods that can robustly define translational regulation from other dynamic RNA processes may help to further disentangle the mechanics of light response in plants (Shirokikh, 2022).

Lastly, it was unclear the extent to which rapid changes in mRNA abundance could contribute to protein‐level changes (Crisp et al., 2017). In general, we found that changes in polysome‐associated mRNA levels correlated with those in the total mRNA pool. This was highlighted by temporal changes measured in previously defined RRGD loci (Figure 5; Figure S6). Therefore, transcript‐level changes in total mRNA should translate to increased ribosome association, resulting in greater translation. Whether or not this would lead to gross changes in protein abundance is unclear, depending on the stability of the protein product (Li et al., 2017). Nonetheless, our observations suggest that the transcriptional compensation observed at genes encoding proteins degraded under high light, should result in elevated translation that contributes to maintaining proteostasis during stress (Li et al., 2022).

## MATERIALS AND METHODS

### Plant growth and treatments

Arabidopsis seeds were sown onto moist soil (Martins Seed Raising and Cutting Mix, Yass, NSW, Australia) supplemented with Osmocote Exact Mini slow‐release fertiliser at 1 g L^−1^ dry volume of soil and 1 L of 0.3% (v/v) AzaMax (Organic Crop Protectants, Padstow, NSW, Australia). Seeds were covered with plastic wrap and stratified at 4°C in the dark for at least 72 h to break dormancy and synchronise germination. Stratified seeds were transferred to a temperature‐controlled Conviron S10H growth chamber (Conviron, Winnipeg, Manitoba, Canada) fitted with a mixture of 250 W metal halide (MH 250 W/U; Venture Lighting, Solon, Ohio, USA) and high‐pressure sodium lamps (SON‐T 250 W E E40 SL/12; Phillips, Amsterdam, Netherlands). Plants were cultivated under a 12‐h photoperiod of 100 μmol photons m^−2^ sec^−1^, 21°C and 55% relative humidity. Plants were watered to saturation the day preceding light stress application. Light stress was induced by increasing the light intensity 10‐fold (i.e. 1000 μmol photons m^−2^ sec^−1^), resulting in a ‘warm’ high light treatment (simulating sunlight, Δ*T* = 7°C) that effectively induces oxidative stress (Crisp et al., 2017; Jung et al., 2013). For recovery, plants were returned to pre‐stress light conditions. Plants were moved between pre‐programmed growth chambers to impose light stress and recovery. All experiments were performed at the midday point of the light period to exclude diurnal effects.

For transcriptional inhibition, cordycepin (3′‐deoxyadenosine; Sigma‐Aldrich, St. Louis, MO, USA) was syringe infiltrated on the abaxial side of fully expanded leaves (true leaves 4–6) of 28‐day‐old plants. Individual leaves were infiltrated with incubation buffer (1 mm PIPES [pH 6.25], 1 mm sodium citrate, 1 mm KCl, 15 mm sucrose) containing 0.6 mm cordycepin (Gutierrez et al., 2002; Seeley et al., 1992), or without (mock), using a 1 ml needleless syringe (Terumo). For each infiltration, the abaxial side of a leaf was pressed gently, yet firmly, against a syringe containing the appropriate buffer, which was then slowly expelled. Leaves were infiltrated until visibly saturated down to the base of the petiole, requiring approximately 100 μl of buffer, in order to achieve a consistent concentration of cordycepin in the apoplastic space between leaves. Once the buffer had permeated through the entire leaf, the syringe was removed and plants were incubated for 10 min (unless otherwise stated) before further treatment and harvesting. Each biological replicate represents leaves from independent plants infiltrated with either mock or cordycepin‐containing buffers. To minimise biological variation, infiltration of the two buffers per biological replicate was performed on separate leaves, from the same plant, at each time point per condition: US, HL or REC. For harvesting, leaves were excised at the base of the petiole and immediately flash‐frozen with liquid nitrogen. Frozen tissue was stored at −80°C until ready for processing.

### RNA isolation

Frozen tissue was ground into a fine powder using a ⅛′′ steel ball bearing with 1 min shaking at 25 Hz in a TissueLyser II (Qiagen, Hilden, Germany). Total RNA was extracted from finely ground tissue using TRIzol reagent (#T9424‐200ML; Sigma‐Aldrich, St. Louis, MO, USA) at a ratio of 1 ml solution per 100 mg ground tissue. Residual phenol was removed from the crude extract through two chloroform extractions at a ratio of 1:5 (v/v), followed by precipitation using isopropanol at 1:1 (v/v). The precipitated RNA was washed twice with 70% ethanol and resuspended in 1 mm sodium citrate buffer (pH 5.4). RNA quantification was performed through spectrophotometric analysis at 260 nm using the Nanodrop ND‐1000 Spectrophotometer, and RNA quality was assessed using 1% agarose gel electrophoresis or the LabChip GX Touch (PerkinElmer, Waltham, MA, USA). Five micrograms of purified total RNA was combined with 5 μl of TURBO DNase buffer and 1 μl TURBO DNase (Thermo Fisher Scientific, Waltham, MA, USA) in a 50 μl reaction and incubated at 37°C for 30 min. DNA nuclease‐treated RNA was then purified using 1.8× Sera‐Mag paramagnetic particles.

### Quantitative reverse‐transcription polymerase chain reaction analysis

DNA‐depleted RNA was reverse transcribed into cDNA using either Invitrogen Superscript III (Thermo Fisher Scientific, Waltham, MA, USA) or Maxima H Minus (Thermo Fisher Scientific, Waltham, MA, USA) reverse transcriptase according to the manufacturer's instructions. For detection of mRNAs, 1 μg of DNA nuclease‐treated RNA was combined with dNTPs and an Oligo(dT)_18_ primer in a 4.5 μl reaction volume to final concentrations of 2.2 mm and 11.1 μm, respectively, and incubated for 5 min at 65°C. For Superscript III based reverse transcription, 1× first strand reaction buffer, 10 mm DTT and 100 units of Superscript III Reverse Transcriptase were added to a final volume of 10 μl before incubation at 50°C for 60 min and 70°C for 15 min. For Maxima H Minus based reverse transcription, 1× reverse‐transcription buffer and 100 units Maxima H Minus Reverse Transcriptase was added to the reaction mix before incubation at 50°C for 30 min and 80°C for 5 min. For the detection of pre‐mRNA, 500 ng of DNA nuclease‐treated RNA was combined with 1.05 mm dNTPs and 10.5 μm random hexamers (Qiagen, Hilden, Germany) in a reaction volume of 14.25 μl before incubation at 65°C for 5 min. 1× First strand reaction buffer, 5 mm DTT and 150 U Superscript III (Thermo Fisher Scientific, Waltham, MA, USA) were added to a final volume of 14.25 μl, before incubation at 25°C for 10 min, 50°C for 1 h, and 70°C for 15 min. Expression of mRNA and pre‐mRNA was then assayed by semi‐quantitative reverse‐transcription PCR (qRT‐PCR) on a Roche LightCycler480 using SYBR Green I (Roche Diagnostics, Florham Park, NJ, USA). Raw fluorescence data were analysed using LinRegPCR to perform background subtraction, determine PCR efficiency and calculate starting concentration (*N*_0_; arbitrary fluorescence units; Ramakers et al., 2003; Ruijter et al., 2009). N_0_ values were used to calculate fold changes for target genes, which were normalised to changes in the housekeeper *PROTEIN PHOSPHATASE 2A SUBUNIT A3* (*PP2AA3*, *AT1G13320*). At least three biological replicates per genotype per time point were sampled, and each qPCR reaction was run in technical duplicate or triplicate. Primer sequences are provided in Table S1.

### mRNA sequencing

The Illumina TruSeq Stranded mRNA Kit was used to prepare strand‐specific (reverse stranded: first read maps to the reverse strand) polyA‐enriched sequencing libraries according to the manufacturer's instructions, except that all reactions were scaled down by one‐third and SuperScript III (Invitrogen) was used for first strand synthesis at 50°C. Libraries were constructed using Illumina unique dual index adapters in a 15‐cycle indexing PCR. All clean‐up steps were performed using nuclease‐free AMPure XP beads (Beckman Coulter, Sharon Hill, PA, USA) or Sera‐mag SpeedBeads (GE Healthcare, Chicago, IL, USA). Library concentration and fragment distribution were determined on the Qubit (double‐stranded DNA high sensitivity kit; Invitrogen) and LabChip GX Touch (DNA High Sensitivity kit; PerkinElmer), respectively. The concentration and peak fragment size were used to determine individual library molarity (assuming the average molar mass of one DNA bp = 650 g mol^−1^). Individual libraries were pooled in equal molar ratios and sequenced on a NextSeq500 (75‐bp single‐end, Table S2) at the ACRF Biomolecular Research Facility (The Australian National University, Canberra, ACT, Australia).

Raw sequencing reads were trimmed to remove adapter sequences and low‐quality base calls (PHRED <20, ‐q 20) with *Trim Galore!* (v0.6.4), a wrapper for *Cutadapt* (v1.18), followed by inspection with *FastQC* (v0.11.8). Trimmed reads were used for transcript quantification using *Kallisto* (Bray et al., 2016). *Kallisto index* (‐k 21) was used to build a transcript index based on TAIR10 coding sequences (Ensembl release 51: Arabidopsis_thaliana.TAIR10.cdna.all.fa.gz; Kersey et al., 2016). Bootstrapped transcript‐level abundances were computed using *Kallisto quant* (‐rf‐stranded ‐‐bias ‐‐single ‐b 10 ‐l 300 ‐s 100). Transcript‐level abundance estimates were summarised to gene‐level counts, using *Tximport* (‘lengthScaledTPM’; Soneson et al., 2015), for genes detected at an abundance of at least 0.5 TPM in more than six *t*_10_ samples (17 256 loci, Table S3). The *RUVr* procedure of the *RUVSeq* package was applied to account for variation between experimental batches (Risso et al., 2014). Briefly, deviance residuals were quantified using a first‐pass generalised linear model of rounded counts, without global scaling, on the covariates of interest. Then, a factor analysis is performed using the deviance residuals to compute batch‐corrected counts using the *RUVr* function. Multidimensional scaling was performed on the batch‐corrected counts using *plotMDS* (gene.selection = “pairwise”). Differential gene expression analysis was performed on batch‐corrected counts using the *edgeR* quasi‐likelihood pipeline without TMM normalisation (Chen et al., 2016). A quasi‐likelihood negative binomial generalised log‐linear model was fit to the adjusted counts (glmQLFit, robust = T) followed by the application of quasi‐likelihood *F*‐tests (glmQLFTest), with FDR correction for multiple hypothesis testing, to detect significant differentially expressed genes (FDR adjusted *P*‐value <0.05).

Adjusted counts were converted to abundance (counts per million, CPM) and used to compute fractional decreases in mRNA abundance (relative to the mean at *t*_10_ per condition). The *t*_10_ time point was shared between HL and REC samples as the same batch of infiltrated plants was either retained under HL or transferred to standard growth conditions for REC. Decay factor normalisation was performed to account for the apparent increase in abundance of relatively stable genes, which constitute a greater proportion of the total RNA pool while those less stable degrade without replacement when transcription is inhibited (Sorenson et al., 2018). To do this, a decay factor was computed for each time point per condition, across the cordycepin‐treated samples, using a set of reference genes. Reference genes were selected based on their abundance (>95th percentile [log_2_ CPM + 0.01]) and variance (<25th percentile [coefficient of variation CPM]) across mock‐infiltrated samples. This produced the following 52 reference genes: *AT4G01050*, *AT4G24280*, *AT5G36790*, *AT1G07920*, *AT5G13650*, *AT1G05850*, *AT1G62750*, *AT4G22890*, *AT3G62030*, *AT3G19170*, *AT2G18960*, *AT3G23400*, *AT5G42270*, *AT3G63140*, *AT3G11630*, *AT4G24770*, *AT1G65960*, *AT3G54050*, *AT4G01150*, *AT5G61410*, *AT1G11860*, *AT4G32260*, *AT5G60600*, *AT5G42980*, *AT3G55800*, *AT3G46780*, *AT3G16140*, *AT4G20360*, *AT3G14420*, *AT1G56070*, *AT5G66190*, *AT3G02470*, *AT1G30380*, *AT4G03280*, *AT2G30950*, *AT5G60390*, *AT4G04640*, *AT1G55670*, *AT3G60750*, *AT1G44575*, *AT5G35630*, *AT4G28750*, *AT1G20020*, *AT3G12780*, *AT5G50920*, *AT1G42970*, *AT1G20340*, *AT4G38970*, *AT4G37930*, *AT4G12800*, *AT5G01530* and *AT3G61470*. The mean fold increase computed across reference genes in cordycepin‐infiltrated samples should reflect the fold change in the total RNA pool. This was applied as a scaling factor on fractional decreases at each time point per condition. Lastly, any genes still displaying a fold increase ≥1.5 were excluded from the analysis, leaving 14 386 and 9339 loci for unstressed and all conditions, respectively.

### mRNA half‐life analysis

Decay factor‐normalised fractional decreases in mRNA abundance were used to compute half‐life (*t*_1/2_) per condition (assuming exponential decay and first‐order kinetics) (Ross, 1995). The decay constant (*k*_d_) was first computed using log‐linear modelling of the change in mRNA abundance as a function of time using individual data points from cordycepin‐infiltrated samples in each condition: *k*_d_ = −log_e_[*C*/*C*_10_]/*dT*. From this, the condition‐specific half‐life of each gene could be solved using: *t*_1/2_ = log_e_(2)/*k*_d_. The *R* package *lme4* was used for building linear mixed‐effects models with fixed (Time) and random variables (experimental batch) (Bates et al., 2015). The conditional *R* ^2^ and accompanying *F*‐statistic and *P*‐value were used to evaluate whether the data fit an exponential decay model (*P* < 0.05) on a gene‐by‐gene basis, with the *R* packages *piecewiseSEM* and *lmerTest* (Kuznetsova et al., 2017; Lefcheck, 2016). Finally, any genes calculated with *k*_d_ < 0 or half‐life >1440 min (1 day) were removed from the analysis. In total, 6711 and 3960 genes could be statistically modelled (*P* < 0.05) under unstressed and all conditions, respectively. Half‐lives calculated under unstressed conditions were directly compared with those reported for a common set of 4497 genes across multiple genome‐wide studies in Arabidopsis (Chantarachot et al., 2020; Narsai et al., 2007; Sorenson et al., 2018; Szabo et al., 2020). The R package *dabestr* was used to compute paired effect size parameters: paired mean difference (*θ*), paired median difference (*μ*_1/2_) and paired Cliff's delta (*δ*) using the functions *mean_diff*, *median_diff* and *cliffs_delta*, respectively, with 10 000 bootstrap resamples. Significant functional enrichments (FDR < 0.05) were assessed using *ShinyGO v0.76.1* (Ge et al., 2020).

### Polysome‐associated mRNA sequencing

Polysome‐associated mRNA sequencing was performed using an adapted protocol (Lecampion et al., 2016), including modifications to the polysome extraction buffer based on Hsu et al. (2016). Briefly, 250 mg of ground plant tissue was dissolved in 1 ml of polysome extraction buffer (160 mm Tris‐Cl [pH 7.6], 80 mm KCl, 5 mm MgCl_2_, 5.36 mm EGTA [pH 8], 0.5% IGEPAL CA‐630, 40 U ml^−1^ RNasin Plus RNase inhibitor [Promega], 150 μg ml^−1^ cycloheximide and 150 μg ml^−1^ chloramphenicol) and incubated on ice for 10 min. Samples were repeatedly centrifuged at 16 000 *g* for 5 min at 4°C until the supernatant was clear. Samples were loaded onto sucrose gradients, consisting of layers of 50% (1.68 ml), 35% (3.32 ml), 20% (3.32 ml) and 20% (1.68 ml), with the two 20% layers added separately. The buffer used in the gradients consisted of 400 mm Tris‐Cl (pH 8.4), 200 mm KCl, 100 mm MgCl_2_, 10.12 μg ml^−1^ cycloheximide and 10.12 μg ml^−1^ chloramphenicol. Gradients were centrifuged at 41 000 rpm with an SW41Ti rotor for 2 h at 4°C. Each gradient was passed through a spectrophotometer from the highest to lowest density and absorbance at 260 nm recorded, with 1 ml fractions collected using a BR‐188 Density Gradient Fractionation System (Brandel). Fractions were pooled into monosomal and polysome sets and extracted using 5:1 acid phenol:chloroform. Brief, an equal volume of the phenol:chloroform mix was added to each fraction, before centrifugation at 16 000 *g* for 10 min. The aqueous layer was extracted a second time using a one‐fifth volume of chloroform, before the RNA was precipitated using an equal volume of isopropanol in addition to sodium acetate (pH 5.2) to a final concentration of 300 mm. The precipitated RNA was washed twice with 70% ethanol and resuspended in a 1 mm sodium citrate (pH 5.4) solution. Purified polysome RNA and total mRNA purified from paired ground tissue (purified as described above) were used to generate sequencing libraries using the Illumina TruSeq Stranded mRNA kit as described above.

Quality control of raw reads was carried out using *FastQC*, with adapter trimming performed using *scythe* (‐p 0.1) and quality trimming performed using *sickle* (‐q 20 ‐l 20). Reads were aligned to the TAIR10 Arabidopsis genome using *subjunc* to report a single unambiguous mapping location per read (Liao et al., 2013). Sorting, indexing and compression were carried out with samtools (Li et al., 2009), and read counts per loci were calculated using *featureCounts* (‐s 2 for reverse stranded libraries; Liao et al., 2014). Polysome‐associated mRNA samples in replicate one were omitted from the following analysis due to limited read depth, leaving two biological replicates. Reads mapping to rRNA were removed before performing TMM normalisation and calculating reads per kilobase of transcript per million mapped reads (RPKM) at the gene level in *edgeR* (Robinson et al., 2010). Log_2_ RPKM values from RNA sequencing of polysome‐associated mRNA (unstressed, 0 min HL) were directly compared with those determined for 25‐day‐old Col‐0 in Carpentier et al. (2020) for genes detected in both datasets at RPKM > 0. Quasi‐likelihood *F*‐tests were performed, as described above, to detect significantly differentially ribosome‐associated transcripts. RPL was calculated on a gene‐specific basis by dividing its abundance (RPKM) in polysome‐associated RNA by that of total RNA for each replicate.

## AUTHOR CONTRIBUTIONS

PAC and BJP conceived the project. ABS, DRG, MM, BJP and PAC designed experiments. ABS and DRG performed *in planta* cordycepin experiments and mRNA sequencing. ABS, MM, YJ and NES performed polysomal RNA sequencing. ABS, DRG and AFB performed data analysis. ABS, DRG and PAC led manuscript preparation. All authors read and commented on the manuscript.

## CONFLICT OF INTEREST

The authors declare no conflicts of interest.

## Supporting information


**Table S1.** Primers used for qRT‐PCR gene expression analyses.
**Table S2.** RNA half‐life sequencing statistics.
**Table S3.** Gene‐level TPM estimates for genes, defined in TAIR10 (Ensembl Release 51), detected at TPM > 0.5 in at least six *t*_10_ samples.
**Table S4.** Differential expression testing in mock and cordycepin‐treated samples during high light stress and recovery.
**Table S5.** mRNA half‐lives determined using log‐linear regression (*P* < 0.05) for genes in Arabidopsis under unstressed conditions.
**Table S6.** Transcripts with reduced half‐life in mature leaves compared with prior published work in cell culture and juvenile seedlings.
**Table S7.** mRNA half‐lives determined using log‐linear regression (*P* < 0.05) in Arabidopsis under unstressed (US), high light (HL) and recovery (REC).
**Table S8.** High‐light destabilised transcripts.
**Table S9.** Polysome‐bound mRNA‐sequencing statistics.
**Table S10.** Gene‐level RPKM values from sequencing polysome‐bound and total mRNA fractions during light stress and recovery.
**Table S11.** Differential gene expression and relative polysomal loading under high light and recovery.
**Table S12.** Genes with increased relative polysome loading during late high light stress (60 versus 30 min of high light).

**Figure S1.** Pre‐mRNA levels decrease more rapidly during recovery than mRNA.

**Figure S2.** RNA decay experiment quality control.

**Figure S3.** Representative RNA decay curves.

**Figure S4.** Detecting in planta changes in mRNA stability between conditions.

**Figure S5.** Rapid recovery gene downregulation is dependent on the length of high light exposure in Arabidopsis.

**Figure S6.** Uncoupling of total and polysome‐bound mRNA abundance within 30 min of recovery from light stress in Arabidopsis.

**Figure S7.** Expression profiles of light‐induced total and polysome‐associated mRNAs during light stress and recovery in Arabidopsis.

## Data Availability

All codes used for analyses are available on GitHub (https://github.com/dtrain16/NGS‐scripts). All sequencing data are accessible at the NCBI's Gene Expression Omnibus (Edgar et al., 2002) at accession number GSE201015 (https://www.ncbi.nlm.nih.gov/geo/query/acc.cgi?acc=GSE201015).
