# Supplementary material for: Dynamics of mRNA fate during light stress and recovery: from transcription to stability and translation
Source: Plant J. 2023 Nov 10;117(3):818–39. doi: 10.1111/tpj.16531 (PMC10952913; doi:10.1111/tpj.16531)
Supplement: Supplementary file 2 — Figure S1. Pre‐mRNA levels decrease more rapidly during recovery than mRNA. [file TPJ-117-818-s003.pdf]

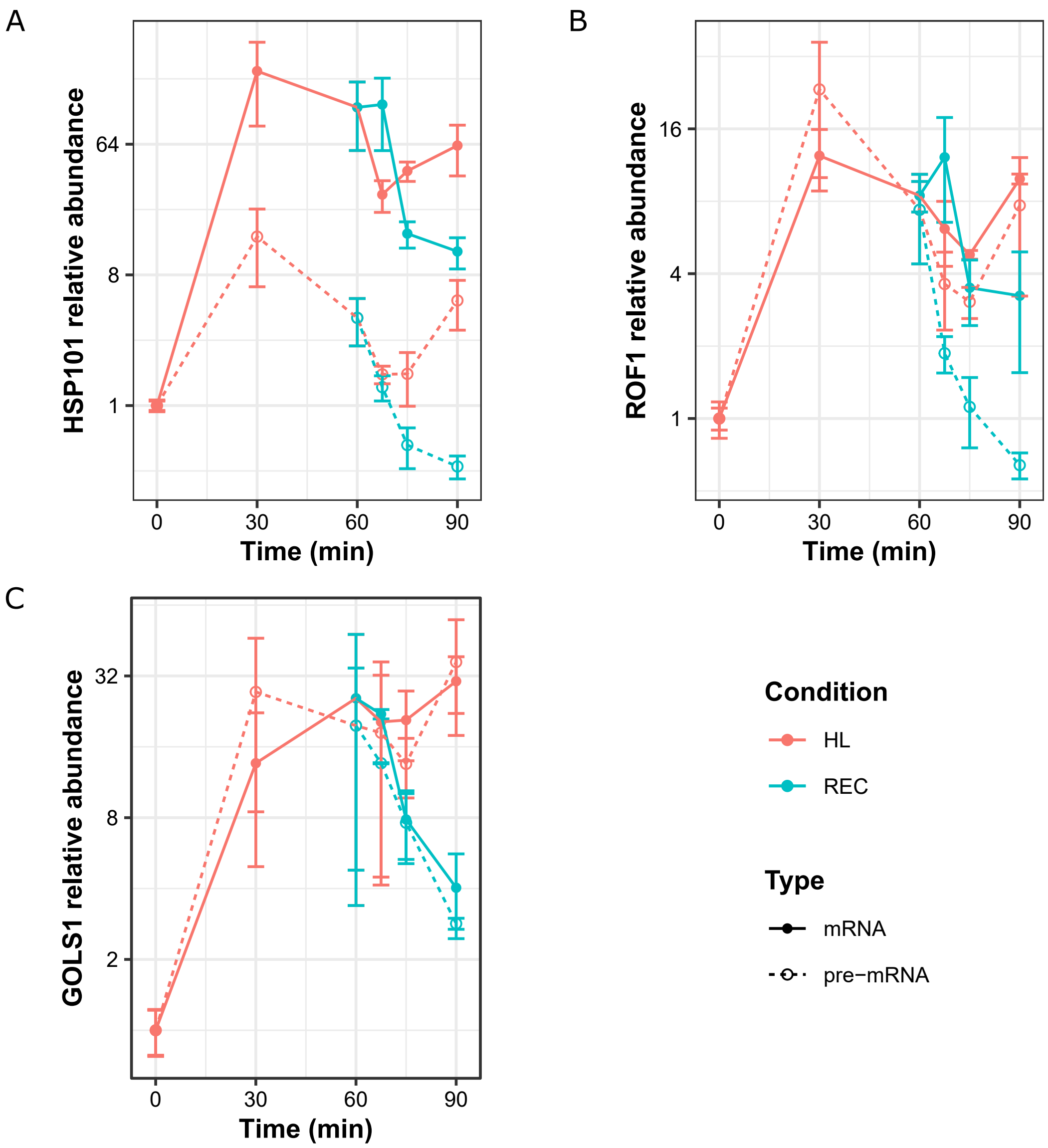

Figure S1. Pre-mRNA levels decrease more rapidly during recovery than mRNA (A-C) Profiles of pre-mRNA and mRNA for *HSP101*, *ROF1*, and *GOLS1* during HL and REC. Data is presented as relative abundance compared to time 0. Points denote means, error bars denote standard error of the mean (n=3).
