# Supplementary material for: Dynamics of mRNA fate during light stress and recovery: from transcription to stability and translation
Source: Plant J. 2023 Nov 10;117(3):818–39. doi: 10.1111/tpj.16531 (PMC10952913; doi:10.1111/tpj.16531)
Supplement: Supplementary file 3 — Figure S2. RNA decay experiment quality control. [file TPJ-117-818-s001.pdf]

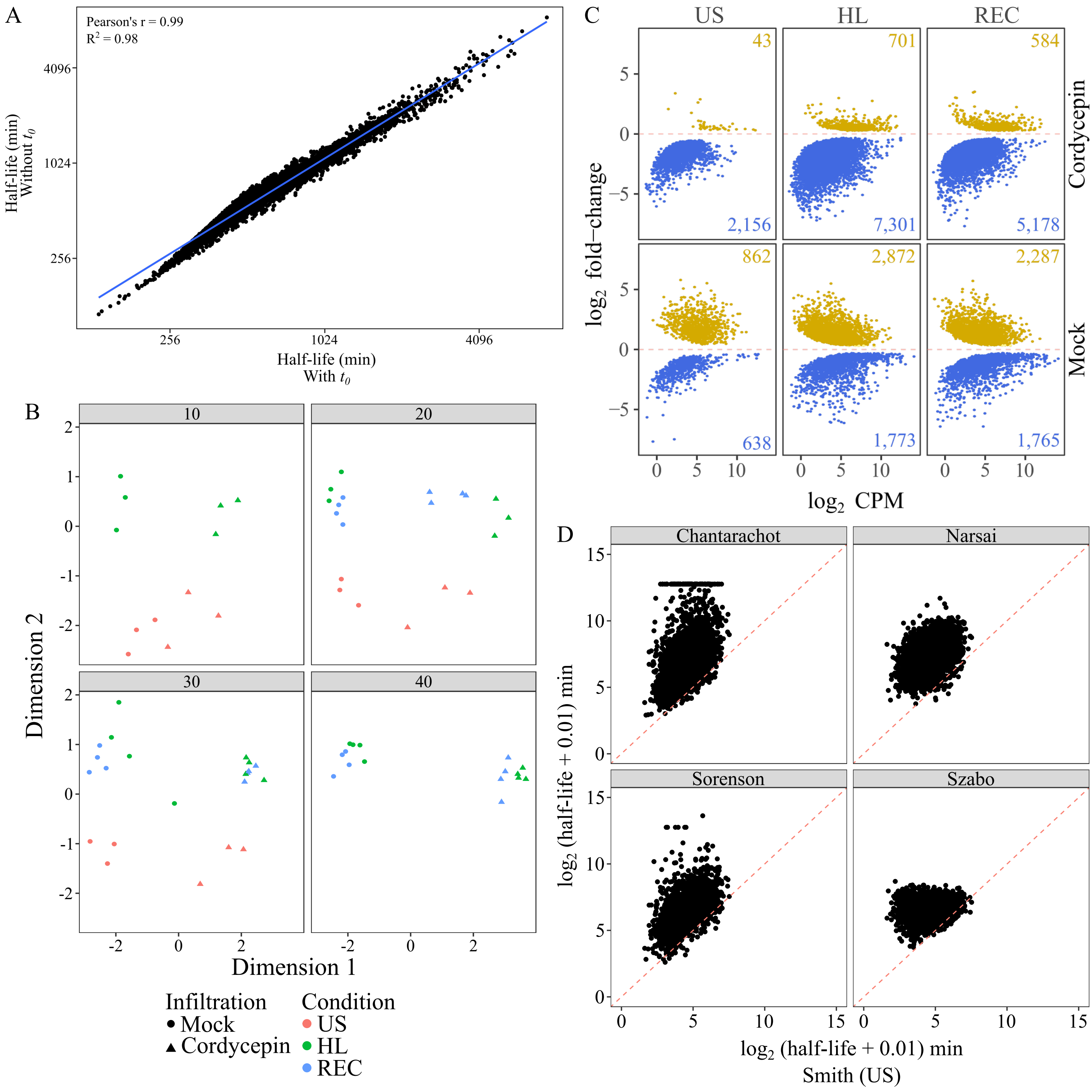

Figure S2. RNA decay experiment quality control
(A) Scatterplot comparing estimates of RNA half-lives computed with log-linear regression on normalized abundances in Col-0 from Sorenson et al. (2018). The x-axis represents the half-life determined using all timepoints. The y-axis represents the half-life calculated without t_0. The blue line represents the fitted linear model used to determine R^2.
(B) Multi-dimensional scaling plot of mock- and cordycepin-treated mRNA sequencing samples where distance reflects the typical $\log_2$ fold-change between samples for the genes that distinguish those samples. Point shapes denote buffer infiltrated (mock or cordycepin) and colour denotes condition (US, HL, or REC).
(C) Differential gene expression between the first and last time points under cordycepin or mock treatment in US, HL, or REC. Up- and down-regulated genes ($p < 0.05$) are denoted by yellow and blue points, respectively.
(D) Scatter plots comparing transcript-specific half-lives (log2 halflife + 0.01) for the 3,960 modelled in thisstudy (x-axis) compared to prior work (y-axis).
