# Supplementary material for: Dynamics of mRNA fate during light stress and recovery: from transcription to stability and translation
Source: Plant J. 2023 Nov 10;117(3):818–39. doi: 10.1111/tpj.16531 (PMC10952913; doi:10.1111/tpj.16531)
Supplement: Supplementary file 4 — Figure S3. Representative RNA decay curves. [file TPJ-117-818-s007.pdf]

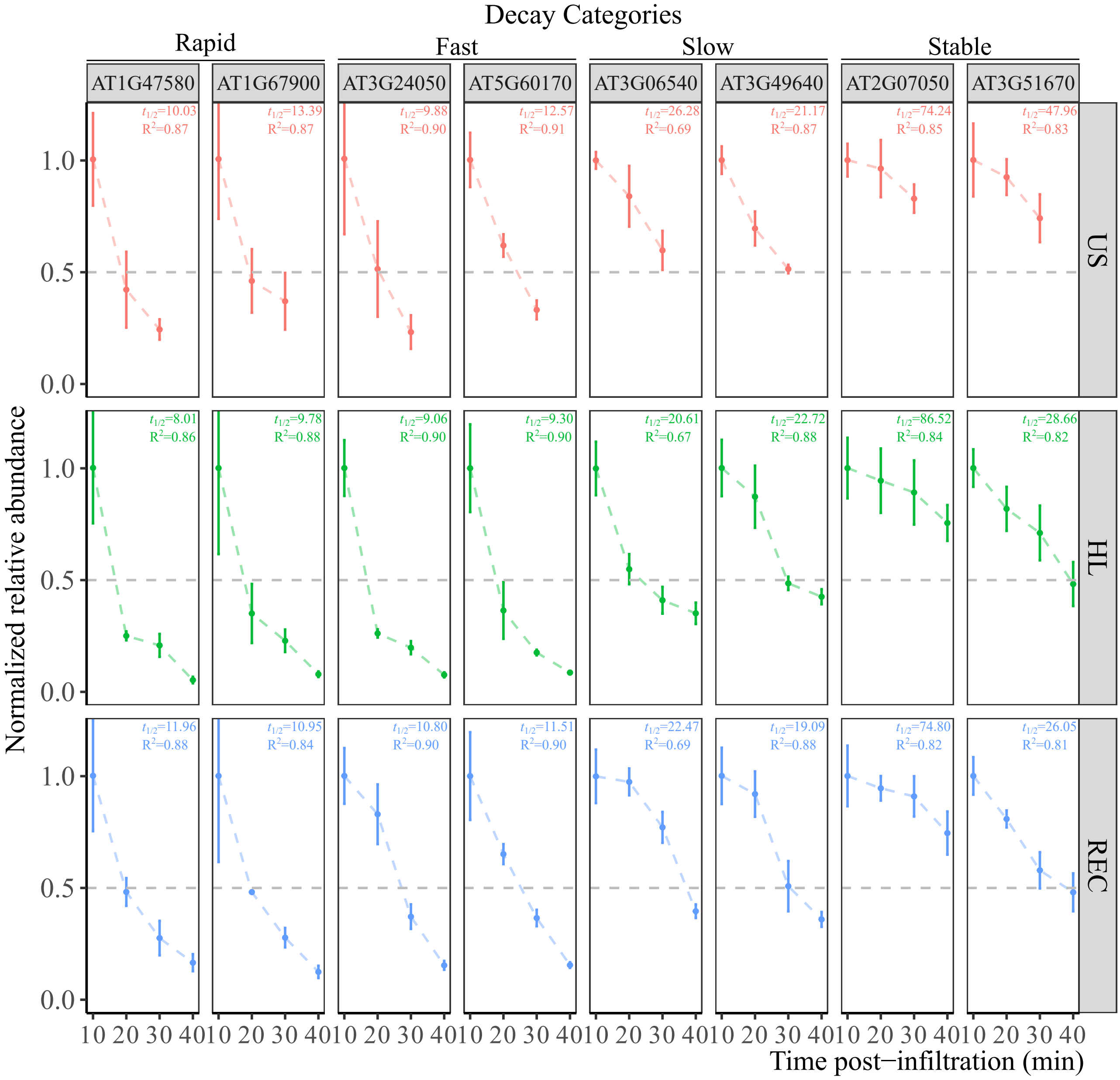

Figure S3. Representative RNA decay curves
Plots represent changes in normalized relative abundance for two example genes from each pattern of decay observed: rapid, fast, slow, stable. Points represent means, error bars represent standard error (n=3-4). Linear mixed-effects modelling was used to determine half-life values and the R-squared obtained for each model is shown.
