# Supplementary material for: Dynamics of mRNA fate during light stress and recovery: from transcription to stability and translation
Source: Plant J. 2023 Nov 10;117(3):818–39. doi: 10.1111/tpj.16531 (PMC10952913; doi:10.1111/tpj.16531)
Supplement: Supplementary file 5 — Figure S4. Detecting in planta changes in mRNA stability between conditions. [file TPJ-117-818-s006.pdf]

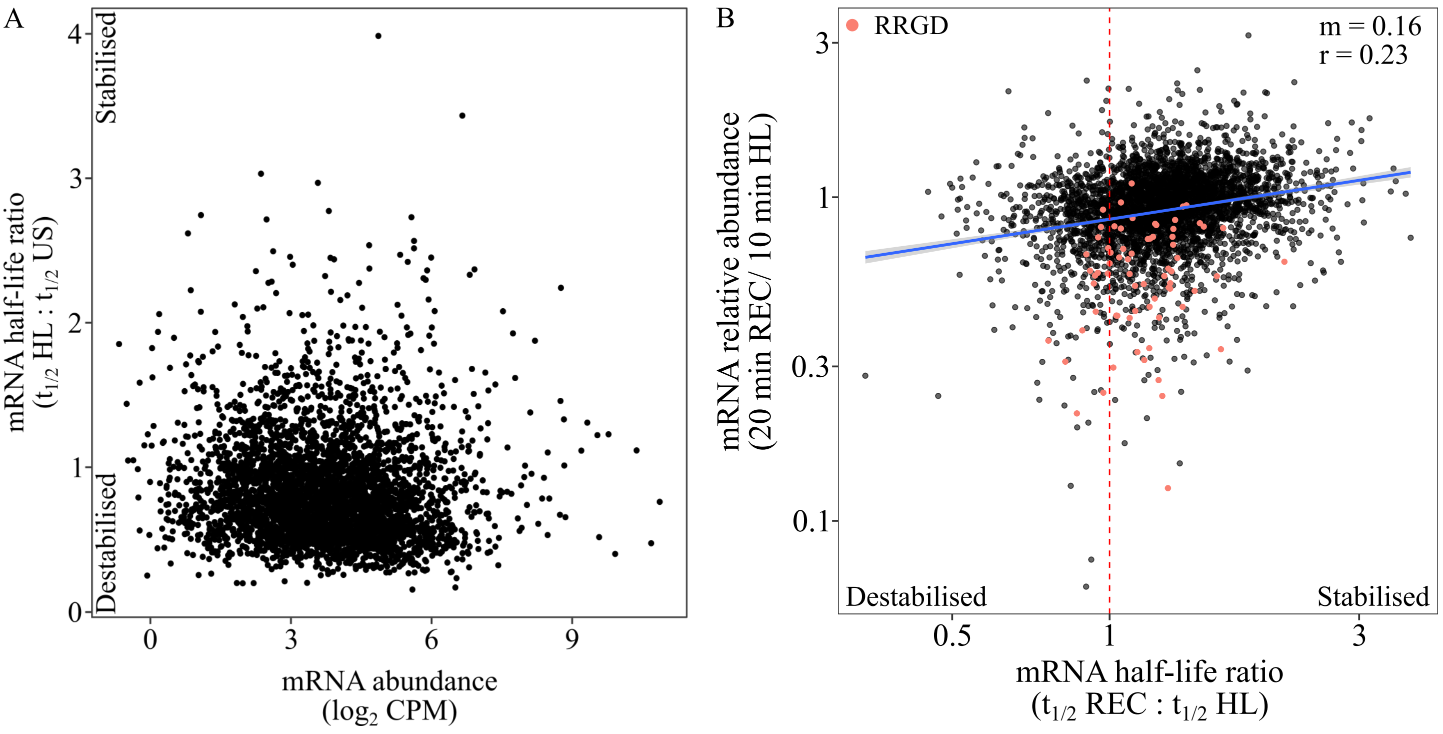

Figure S4. Detecting *in planta* changes in mRNA stability between conditions
(A) Scatter plot relating the half-life ratio (HL/US) to mean mRNA abundance for the 3,960 modelled transcripts. Points represent individual genes.
(B) Scatter plot presenting the fold-change in mRNA abundance (20 min REC/ 10 min HL), in mock-treated samples, versus the half-life ratio (REC/HL) determined for 3,960 modelled transcripts. Points denote individual genes, line denotes fitted linear model, 'm' denotes regression coefficient, and 'r' denotes Pearson's correlation coefficient.
