# Supplementary material for: Dynamics of mRNA fate during light stress and recovery: from transcription to stability and translation
Source: Plant J. 2023 Nov 10;117(3):818–39. doi: 10.1111/tpj.16531 (PMC10952913; doi:10.1111/tpj.16531)
Supplement: Supplementary file 6 — Figure S5. Rapid recovery gene downregulation is dependent on the length of high light exposure in Arabidopsis. [file TPJ-117-818-s004.pdf]

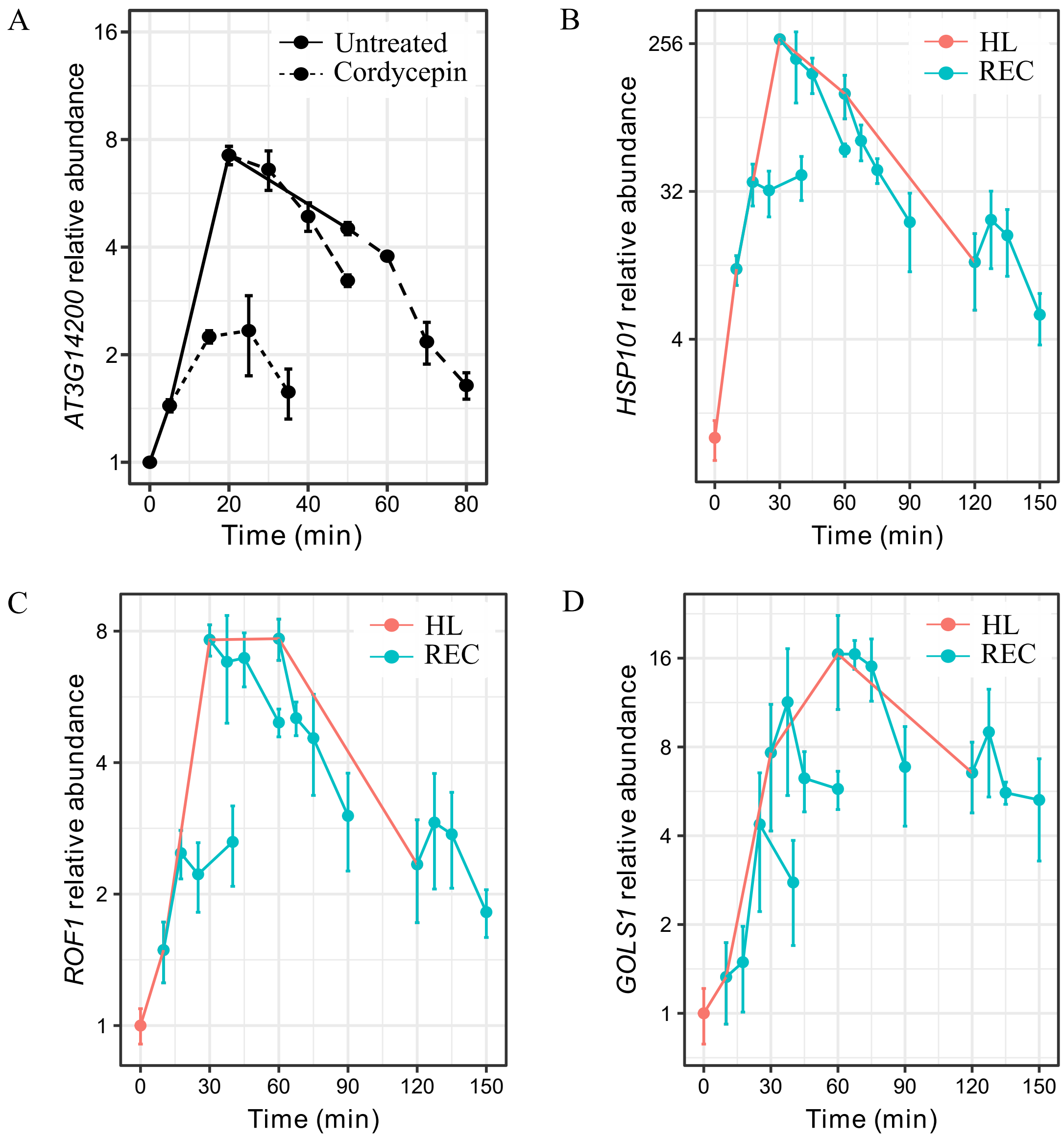

Figure S5. Rapid recovery gene down-regulation is dependent on the length of high light exposure in Arabidopsis

(A) Gene expression changes in *AT3G14200* following cordycepin infiltration carried out at 5, 20, and 50 minutes HL. Solid line denotes untreated leaves, dashed line denotes cordycepin-infiltrated samples.

(B-D) Gene expression profiles of *HSP101*, *ROF1*, and *GOLS1* during REC after various lengths of HL. Red lines denote HL, blue lines denote REC (initiated at 10, 30, 60, and 120 minutes). Points denote means, error bars denote standard error of the mean (n = 3).
