# Supplementary material for: Dynamics of mRNA fate during light stress and recovery: from transcription to stability and translation
Source: Plant J. 2023 Nov 10;117(3):818–39. doi: 10.1111/tpj.16531 (PMC10952913; doi:10.1111/tpj.16531)
Supplement: Supplementary file 7 — Figure S6. Uncoupling of total and polysome‐bound mRNA abundance within 30 min of recovery from light stress in Arabidopsis. [file TPJ-117-818-s002.pdf]

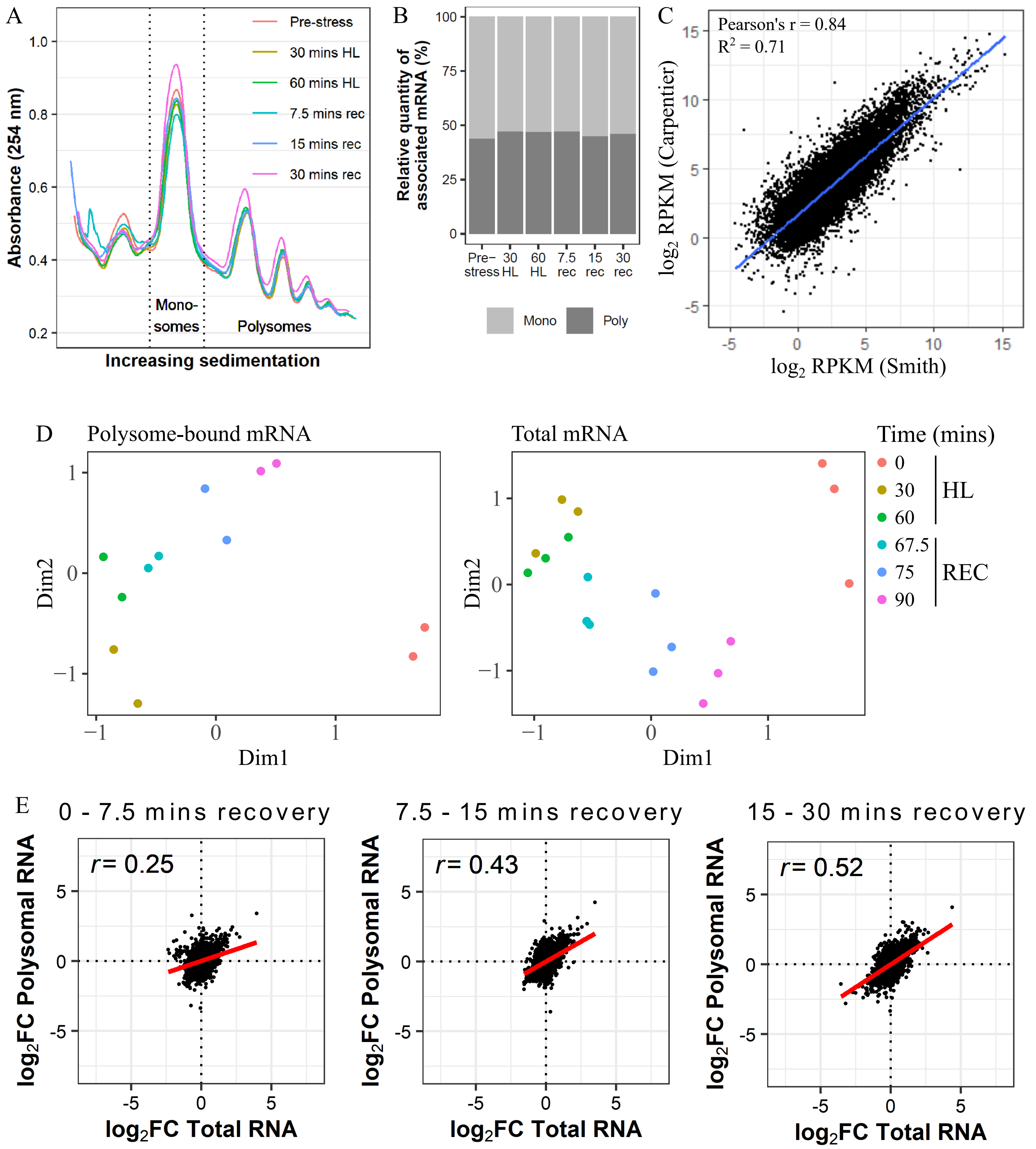

Figure S6. Uncoupling of total and polysome-bound mRNA abundance within 30 minutes of recovery from light stress in Arabidopsis

(A) Monosomes and polysomes extracted from Arabidopsis seedlings were separated using sucrose gradient centrifugation and quantified by reading gradient absorbance profiles at 254 nm light.

(B) Relative abundance of monosomes and polysomes was calculated by integration of the area under the respective sections of the absorbance profile.

(C) Correlation of $\log_2$ RPKM values obtained here with those determined for 25-day old Col-0 in Carpentier et al (2020) for genes detected at RPKM > 0.

(D) Multi-dimensional scaling plots of polysome-bound and total mRNA sequencing samples, during a light stress and recovery timcourse. Distance reflects the typical $\log_2$ fold-change between samples for the genes that distinguish those samples.

(E) Correlation between changes in total and polysome-bound mRNA abundance between recovery time points. r denotes Pearson's correlation coefficient.
