# Supplementary material for: Dynamics of mRNA fate during light stress and recovery: from transcription to stability and translation
Source: Plant J. 2023 Nov 10;117(3):818–39. doi: 10.1111/tpj.16531 (PMC10952913; doi:10.1111/tpj.16531)
Supplement: Supplementary file 8 — Figure S7. Expression profiles of light‐induced total and polysome‐associated mRNAs during light stress and recovery in Arabidopsis. [file TPJ-117-818-s005.pdf]

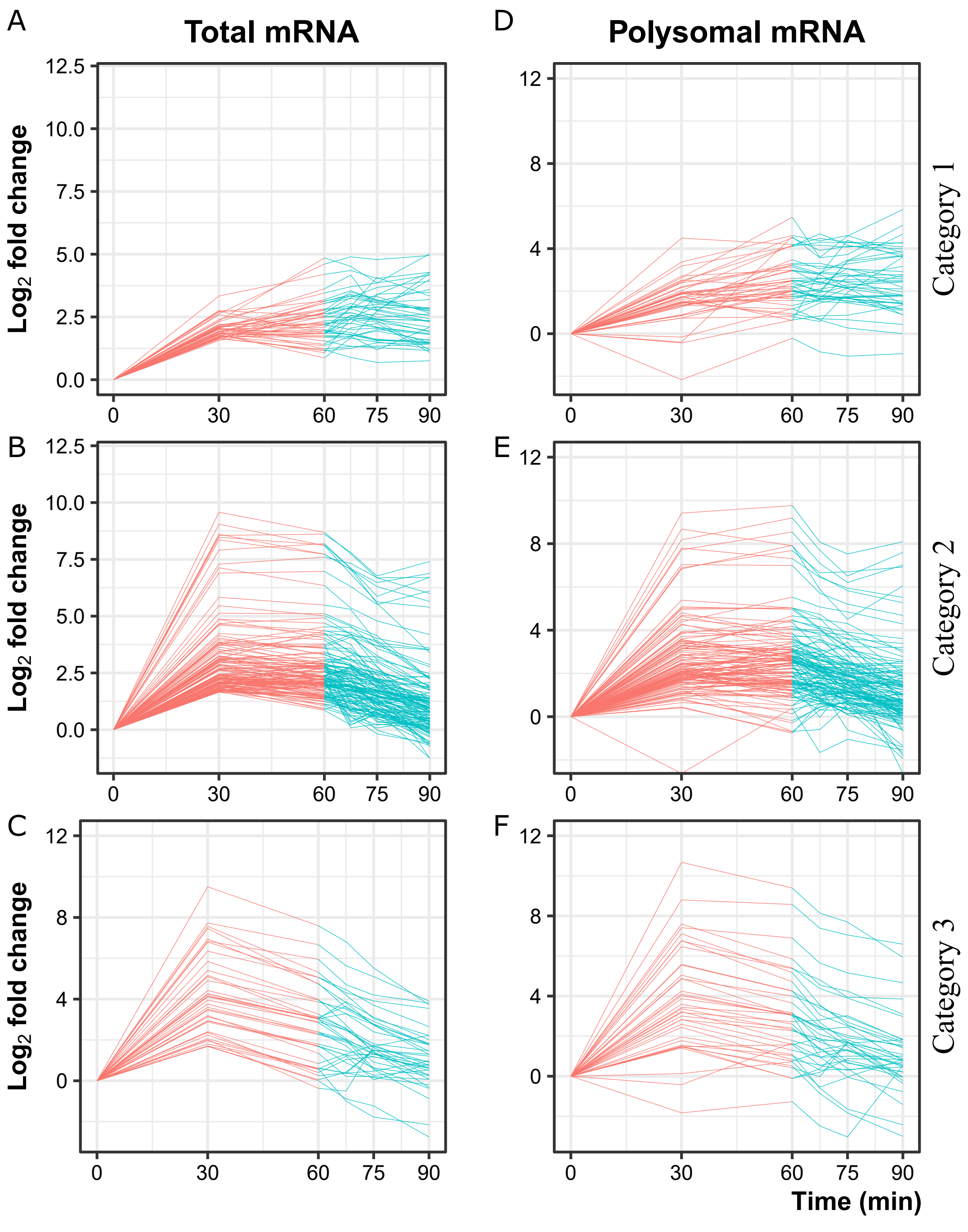

Figure S7. Expression profiles of light-induced total and polysome-associated mRNAs during light stress and recovery in Arabidopsis

(A-C) Genes induced by HL (FC > 3 after 30 mins) were categorised by their expression profiles in the total mRNA fraction. Category 1 genes remained elevated during REC. Category 2 genes were significantly down-regulated during REC. Category 3 genes were significantly down-regulated between 30 and 60 minutes HL (n=3).

(D-F) The corresponding changes in polysome-bound mRNA are displayed (n=2).

Lines represent mean relative abundance over time during HL (red) and REC (blue).
